# Embodied intelligence-driven adaptive collaboration in supply chains: A four-dimensional synergy framework and mechanism analysis

**DOI:** 10.1371/journal.pone.0351058

**Published:** 2026-06-09

**Authors:** Ziqiao Ding, Hanjiang Lin, Huiying Xu, Xiaolei Zhang, Xinzhong Zhu

**Affiliations:** 1 Zhejiang Key Laboratory of Intelligent Education Technology and Application, Jinhua, China; 2 School of Computer Science and Technology of Zhejiang Normal University, Jinhua, China; Beijing Technology and Business University, CHINA

## Abstract

Existing research focuses on data-driven algorithm optimization but overlooks the embodied nature of supply chains as physical and digital integrated systems, leading to a disconnect between AI and physical collaboration. This study introduces embodied intelligence into supply chain management, transcending the traditional paradigm to propose an adaptive collaboration framework through embodied perception, contextual reasoning, and physical execution. It deconstructs the core of supply chain embodied intelligence, revealing issues such as fragmented perception and delayed feedback. Based on embodied cognition and complex adaptive systems theory, a four-layer architecture with embodied perception, contextual reasoning, physical execution, and closed-loop feedback is constructed, clarifying its mechanisms. Future directions in theory, technology, and practice are outlined. This work deepens the integration of embodied intelligence with supply chains, bridges the digital and physical divide, and advances supply chain management toward an embodied adaptive paradigm for next-generation intelligent systems.

## 1. Introduction

### 1.1. Research background

Global supply chains are facing unprecedented shocks of dynamism, complexity, and uncertainty: personalized consumer demands have shortened product iteration cycles, geopolitical conflicts have caused fluctuations in the supply of key materials, and emergencies such as extreme weather have triggered frequent disruptions to logistics networks [1–3]. These changes place extremely high demands on the real-time response speed and dynamic adaptability of supply chains, making traditional supply chain management models relying on manual coordination and static planning increasingly inadequate [4]. More broadly, sectoral transformations exemplify how societal shifts continuously reshape supply-demand landscapes. For instance, the rapid advancement of intelligent education has driven surging demand for online learning terminals, diversified smart teaching equipment, and sustained pressure on computing infrastructure supply. These domain-specific dynamics, combined with the macro-level disruptions discussed above, collectively underscore the necessity for supply chains to possess real-time adaptive capabilities rather than relying on static planning models.

Although artificial intelligence technologies have demonstrated application value in supply chain demand forecasting [5], inventory optimization [6], and path planning [7]. For instance, deep learning-based models provide accurate demand forecasts for new energy vehicle supply chain enterprises [8], helping optimize production plans and reduce costs. However, traditional intelligent applications adopt a disembedded model that abstracts supply chains into data models for algorithm optimization, ignoring the essential attribute of supply chains as physical-digital integrated systems. Specifically, this model fails to consider the spatial constraints of physical logistics (e.g., warehouse shelf height limits, transportation route width), the contextual dependence of human-machine collaboration (e.g., the impact of workers’ operating habits on equipment scheduling), and the physical interaction characteristics of supply chain nodes (e.g., time window requirements for cargo loading and unloading), resulting in a significant collaboration gap between digital decision-making and physical execution. For instance, many order picking optimization studies only focus on improving the efficiency of the order picking operation itself [9], while failing to form effective collaboration with other interacting processes such as subsequent packaging and distribution scheduling.

As a cutting-edge direction in artificial intelligence, embodied intelligence’s core idea is that intelligent agents generate intelligent behaviors through real-time interaction with the physical environment, dynamic situational perception, and embodied execution, rather than relying on pre-set digital models and offline data. This characteristic is highly consistent with the integrated nature of supply chains involving physical flow and digital decision-making. For example, collaboration between physical operations and digital decisions can be achieved through flexible rolling wheels, real-time tactile feedback, and closed-loop adjustment designs [10]. However, current research on embodied intelligence mainly focuses on technical fields such as robotics (e.g., adaptive grasping of industrial robotic arms) [11,12] and computer vision (e.g., training robots’ spatial reasoning capabilities) [13], and has not yet formed systematic integration with supply chain management theories. It thus cannot provide a complete theoretical support and technical path for solving the adaptive collaboration problems of physical supply chains. Therefore, there is an urgent need to introduce embodied intelligence theory into supply chain management, reconstruct the theoretical system and technical framework of intelligent supply chains, and achieve deep collaboration between digital technologies and physical supply chains.

### 1.2. Research significance

#### 1.2.1. Theoretical significance.

The theoretical contributions of this study are reflected in three dimensions: First, in contrast to existing literature that treats artificial intelligence as an abstract optimization tool applied to supply chain data models [14–16], this study is among the first to systematically introduce embodied intelligence theory into the field of supply chain management. By clarifying the core concept, connotative boundaries, and characteristic dimensions of supply chain embodied intelligence, it fills the theoretical gap in intelligent supply chain collaboration for physical-digital integrated scenarios. This fundamentally shifts the research bias from a digital-above-all approach to a balanced physical-digital fusion perspective. Second, it breaks through the one-way, data-driven paradigm of traditional intelligent supply chains. While prior research has focused on information sharing or digital twin-based mirror simulation, this study constructs a bi-directional adaptive collaboration mechanism of ‘embodied perception-contextual reasoning-physical execution-closed-loop feedback’. This mechanism integrates the agent co-evolution logic of complex adaptive system theory with the contextual interaction logic of embodied cognition theory into supply chain management, crucially repositioning physical execution not merely as an output but as a generative source of intelligence that dynamically feeds back into decision optimization. This enriches the application scenarios and theoretical depth of complex adaptive system theory in the supply chain field. Third, by deconstructing the inherent logic of embodied intelligence-driven supply chain collaboration, this study identifies three core mechanisms including contextual coupling, subject collaboration, and evolutionary optimization, and further provides mathematical formalizations for each mechanism. It lays a solid theoretical foundation for the paradigm transformation of supply chain management from digital optimization to embodied adaptability, and advances the development of supply chain management theory toward in-depth physical-digital integration. The formal definitions and mathematical descriptions of the above mechanisms also support rigorous theoretical verification and facilitate subsequent empirical research.

#### 1.2.2. Practical significance.

The practical value of this study focuses on providing actionable, structured guidance for enterprises to build next-generation intelligent supply chains, distinctly different from current piecemeal technology adoption approaches:

First, it helps enterprises break the persistent separation between digital technologies and physical operations. Rather than deploying technologies in isolation, this study provides an integrated framework for realizing contextual perception and physical execution through the systematic deployment of embodied intelligent equipment (e.g., multimodal sensors, autonomous mobile robots) and the construction of a four-layer collaborative architecture with clearly defined inter-layer interaction protocols.

Second, it guides enterprises to shift from a local mindset of optimizing individual links through algorithms to a systematic mindset of end-to-end embodied collaboration. By specifying the indicator system and implementation path for each architectural layer, the framework enables enterprises to measure and enhance supply chain adaptability to dynamic environments in a structured manner.

Third, it provides specific, theoretically grounded technical paths for the intelligent upgrading of physical supply chain scenarios such as logistics sorting, intelligent warehousing, and flexible production, with each path mapped to corresponding technologies and performance indicators.

### 1.3. Research methods and ideas

This study adopts a combination of interdisciplinary research and logical deduction methods, integrating core ideas from four academic fields: embodied cognition theory, complex adaptive system theory, robotics, and supply chain management, to conduct systematic research.

The research follows a logical thread of identifying theoretical gaps, deconstructing core concepts, constructing frameworks, analyzing mechanisms, and proposing future prospects: Step 1, through a systematic review of existing research on intelligent supply chains and embodied intelligence, identify three core research gaps in traditional intelligent supply chains: theoretical integration, collaboration mechanisms, and paradigm innovation. This clarifies the entry point and research value of this study. Step 2, based on embodied cognition theory and supply chain characteristics, deconstruct the core connotation of supply chain embodied intelligence, extract its characteristics from four dimensions (perception, reasoning, execution, feedback), and define its essential differences from traditional intelligent supply chains. Step 3, with embodied cognition theory as the core, integrate the technical logic of complex adaptive system theory and robotics to construct a theoretical framework for embodied intelligence-driven adaptive collaboration in supply chains, clarifying the functional positioning, technical support, and data interaction relationships of each layer. Step 4, deeply analyze the operational mechanism of the framework, revealing how the three core mechanisms (contextual coupling, subject collaboration, evolutionary optimization) promote adaptive collaboration in supply chains. Step 5, combined with the current research status and industrial practice needs, propose future research directions from three aspects: theoretical expansion, technology integration, and practical implementation, providing references for subsequent studies.

## 2. Literature review and theoretical foundations

### 2.1. Literature review

#### 2.1.1. Research status of intelligent supply chains.

Existing research on intelligent supply chains has formed two main technical application directions, both with significant limitations:

The first type focuses on the application of technologies such as machine learning, digital twins, and big data in optimizing individual supply chain links [14–16]. Deep learning-based demand forecasting models [17] extract patterns from large datasets to improve prediction accuracy, while reinforcement learning-based logistics path planning algorithms [18] optimize transportation routes through iterative training to reduce logistics costs. However, such research generally regards supply chains as data collections, ignoring their physical entity attributes. For example, the model in [19] does not consider product shelf life or priority differences (e.g., replenishment priorities for fresh produce vs. daily necessities), assumes known sales and loss forecasts, and fails to integrate an end-to-end prediction-optimization framework, resulting in poor adaptability in fresh produce supply chain scenarios.

The second type focuses on collaboration mechanisms, exploring information sharing, benefit coordination, and risk-sharing among supply chain nodes. For instance, based on evolutionary game theory, a tripartite evolutionary game model is constructed by incorporating the government and upstream and downstream supply chain enterprises into a unified analytical framework [20]. Through incentive mechanisms such as government subsidies, it reduces the cost of enterprise data sharing, promoting supply chain enterprises to open and share data resources such as orders and production plans, breaking data silos, optimizing supply chain decision-making and resource allocation, and improving collaboration efficiency. However, such research does not consider the deep integration of intelligent technologies and physical execution, failing to solve the disconnection between digital decision-making and physical operations. For example, the study in [21] only achieves the interconnection of order data and sales data, without synchronously integrating physical execution data such as warehouse equipment status (e.g., shelf load, sorter efficiency) and logistics vehicle positions, leading to frequent adjustments of production plans generated by enterprises based on the platform due to logistics delays.

Furthermore, recent studies have shown that supply chain financing can alleviate the financial constraints of small and medium-sized enterprises, and blockchain technology can optimize supply chain financing strategies. These studies also point out that large language models, with their unique natural language processing capabilities, have broad application prospects in supply chain financing decision-making. Specifically, relevant research has innovatively introduced large language models to explore their application in blockchain-driven supply chain financing platforms, and found that the excellent information processing capabilities of large language models can significantly improve users’ financing efficiency. This line of research highlights the application potential of large language models in this field and provides decision-making reference for managers [22]. Such developments highlight the broader trend of AI integration across supply chain functions, yet they remain primarily focused on digital layer optimization without addressing the physical-digital integration challenge central to this study.

Overall, existing research has not broken through the traditional thinking of digital technologies serving supply chain optimization, lacking theoretical exploration of the essential physical-digital integration of supply chains, and thus struggling to support physical supply chains in meeting the demand for adaptive collaboration in dynamic environments.

#### 2.1.2. Research status of embodied intelligence.

The theoretical origin of embodied intelligence can be traced back to the embodied cognition theory proposed in [23], which subverts the traditional view that cognition only occurs in the brain and puts forward the core idea that intelligence arises from the interaction between the body and the environment. In the field of artificial intelligence, embodied intelligence research has formed clear technical directions, mainly focusing on three areas: robotic embodied perception, contextual reasoning, and physical execution. All of these areas emphasize real-time interaction between intelligent agents and the physical environment.

In terms of robotic embodied perception, [24] proposes a general method for autonomous robotic assembly that combines visual and intrinsic tactile perception to continuously track parts within a single Bayesian framework. Based on this, object-centric assembly skills guided by estimated part poses can be realized, even when the visual system is blocked by obstacles. In contextual reasoning, the hierarchical path planning algorithm proposed in [25] relies on the high-precision tracking capabilities of vision and lidar to enable robots to plan efficient and compliant paths in dynamic human crowds, reaching destinations without causing discomfort to others. In physical execution, [26] proposes a deep reinforcement learning-based zero-shot coverage path planning (CPP) framework for mobile robots, achieving zero-shot generalization to different map sizes, sensor configurations, and task types without retraining through designing observation spaces adapted to different map sizes, action masking mechanisms ensuring safety and completeness, size-invariant value functions, and combining curriculum learning and environment randomization.

Despite significant progress in technical research, existing studies have not yet systematically integrated the core ideas of embodied intelligence with supply chain management theories, failing to explain key issues such as how digital decision-making accurately drives physical execution and how physical execution feedback dynamically optimizes digital decision-making in supply chain systems. This results in the application of embodied intelligence technologies in supply chain scenarios remaining scattered and lacking systematic solutions.

#### 2.1.3. Research status of cross-disciplinary integration between embodied intelligence and supply chain management.

Currently, cross-disciplinary research between embodied intelligence and supply chain management is still in its infancy, with scattered achievements and no systematic research system. Existing cross-disciplinary studies mainly focus on two directions: First, the isolated application of embodied intelligence-related technologies (e.g., autonomous mobile robots, multimodal sensors) in individual supply chain links, such as adaptive grasping of warehouse robots and dynamic path planning of logistics vehicles, without constructing an embodied collaboration architecture at the supply chain system level, which remains a local application of technical tools [27,28]. Second, some studies mention the physical-digital integration needs of supply chains but fail to propose systematic solutions combining core embodied intelligence theories (e.g., embodied cognition, closed-loop feedback), remaining at the preliminary conceptual exploration stage.

Overall, existing cross-disciplinary research lacks deep integration of embodied intelligence theory and supply chain management theory, fails to solve the disconnection between digital decision-making and physical execution, and has not formed a complete theoretical framework and technical path for embodied intelligence-driven adaptive collaboration in supply chains. This thus provides clear innovation space for this study. It is worth noting that complementary research streams, such as the integration of large language models with blockchain for supply chain finance [22], although not directly addressing embodied intelligence, point toward a broader recognition of the need for more sophisticated, trustworthy, and intelligent supply chain systems. These parallel developments underscore the timeliness and necessity of our work in bringing embodied intelligence as a unifying framework to bridge digital intelligence with physical operations.

#### 2.1.4. Research gaps.

Based on a comprehensive review of existing research, there are three core research gaps in the field:

First, the theoretical integration gap. Embodied intelligence theory and supply chain management theory have not yet formed systematic integration. Existing research either focuses on the technical development of embodied intelligence or the digital optimization of supply chains, lacking an embodied intelligence theoretical framework tailored to the physical-digital integration characteristics of supply chains, which cannot provide theoretical guidance for the intelligent transformation practice of physical supply chains.

Second, the collaboration mechanism gap. Traditional intelligent supply chain research has not constructed a closed-loop collaboration mechanism of ‘perception-reasoning-execution-feedback.’ Digital decision-making and physical execution operate independently, resulting in insufficient supply chain adaptability and difficulty in coping with dynamic and complex market environments.

Third, the paradigm innovation gap. Existing research remains stuck in the data-driven algorithm optimization paradigm, simplifying supply chains into data models for abstract optimization, ignoring their physical entity attributes and contextual dependence, and failing to achieve a paradigm breakthrough from digital optimization to embodied adaptability in supply chain management, which cannot meet the essential demand for physical-digital integration of supply chains.

### 2.2. Theoretical foundations

#### 2.2.1. Embodied cognition theory.

Embodied cognition theory was first systematically proposed in (Varela, Thompson and Rosch 1991). Its core view is that cognitive processes do not occur solely in the brain but originate from dynamic interactions between the body and the environment. The interaction between the body’s sensory organs, motor systems, and the environment is the core carrier for the formation and development of cognition. This theory provides the core logic for the construction of supply chain embodied intelligence: the intelligent collaboration of supply chains should not rely solely on abstract reasoning of digital algorithms but should achieve contextual perception and decision-making through embodied interactions between physical entities (e.g., logistics robots, multimodal sensors, intelligent warehouse equipment) and the environment. For example, in the traditional model, warehouse robots determine shelf positions only based on pre-set digital maps and cannot accurately locate shelves if they are slightly displaced due to cargo stacking deviations. In contrast, based on embodied cognition theory, warehouse robots need to dynamically adjust grasping paths by integrating shelf layouts captured by visual sensors, cargo weights perceived by tactile sensors, and spatial positioning capabilities of their own motor systems. Even if shelves are slightly displaced, accurate operations can be achieved through multimodal data fusion.

#### 2.2.2. Complex adaptive system theory.

Complex adaptive system theory [29] points out that complex systems consist of a large number of adaptive agents. These agents continuously adjust their behavioral rules through dynamic interactions with the environment and other agents, thereby promoting the evolution and optimization of the system as a whole. As a typical complex adaptive system, the interaction behaviors of supply chain agents, including node enterprises (suppliers, manufacturers, distributors), logistics equipment (AGV robots, sorters), and staff, directly determine the collaboration efficiency and adaptability of supply chains.

In traditional supply chains, interactions between agents mostly rely on manual coordination and delayed system docking, resulting in low collaboration efficiency. For example, when a supplier experiences delays in raw material supply, manufacturers need multiple rounds of communication to confirm the delay time before adjusting production plans [30]. This process takes hours and is prone to production disruptions. In contrast, in embodied intelligent supply chains, embodied intelligent agents (e.g., logistics robots, intelligent sensors) can quickly adapt to environmental changes through real-time data interaction and behavioral collaboration [31]. For instance, in a logistics congestion scenario, after a delivery robot perceives congestion through road condition sensors, it can immediately share road condition data with warehouse robots [32]. Based on this data, warehouse robots promptly adjust the order of cargo sorting, prioritizing delivery orders for non-congested routes to avoid cargo accumulation. Meanwhile, the sorting system synchronously adjusts its sorting rhythm to ensure collaboration with the delivery link. This real-time interaction between agents achieves the overall optimization of the supply chain system.

This theory provides theoretical support for analyzing the interaction mechanisms and system evolution laws of supply chain agents driven by embodied intelligence, helping to understand key issues such as why the collaboration of multiple embodied intelligent agents can enhance supply chain adaptability and how agent interactions promote the evolution of supply chain systems.

#### 2.2.3. Embodied intelligence theory in robotics.

Robotics is the core field for the practical application of embodied intelligence technologies. Embodied intelligence theory in this field emphasizes that robots need to perceive the environment through sensors, interact with the environment through actuators, and continuously optimize decisions based on interaction feedback. Its core characteristics can be summarized as situational perception, autonomous decision-making, and physical execution, with the core logic being a closed-loop cycle of ‘perception-decision-execution-feedback.’ Through feedback data generated by physical interactions, the intelligence level and operational accuracy of robots are continuously improved.

This theory provides direct reference for the technical implementation path of supply chain embodied intelligence, clarifying that supply chain embodied intelligence needs to construct a closed-loop architecture of ‘perception-decision-execution-feedback’ and how to optimize collaboration strategies through physical execution feedback. For example, in logistics delivery scenarios, path planning algorithms and grasping force parameters are optimized through execution data of delivery robots (e.g., delivery time efficiency, cargo damage rate).

## 3. Core connotation and characteristic dimensions of supply chain embodied intelligence

### 3.1. Core connotation

Supply chain embodied intelligence refers to an intelligent model in which supply chain systems achieve real-time interaction with dynamic environments through embodied perception of physical entities (e.g., multimodal sensors, autonomous mobile robots, intelligent sorting equipment), contextual intelligent reasoning, and precise physical execution, and continuously optimize collaboration strategies through closed-loop feedback. As shown in Table 1, its core connotation can be systematically summarized from three dimensions: embodiment, contextuality, and closed-loop.

**Table 1:** Core Connotation of Supply Chain Embodied Intelligence.

Core Connotation	Core Definition	Typical Practical Example
Embodiment	Intelligence originates from direct interactions between physical entities and the environment, rather than solely relying on abstract algorithms and offline data	Intelligent sorting equipment dynamically adjusts sorting rhythm by integrating visual data, weight sensor signals, and real-time conveyor belt speed to avoid cargo accumulation
Contextuality	Decisions need to adapt to specific physical scenario constraints, dynamically adjust with situational changes, and reject one-size-fits-all solutions	Prioritize delivery orders for distant unobstructed routes during logistics congestion, and prioritize nearby orders when logistics is smooth to improve overall efficiency
Closed-loop	Continuously optimize perception models and reasoning algorithms through real-time feedback of physical execution results to achieve dynamic evolution	After a robotic arm damages fragile goods due to excessive grasping force, feedback data drives the model to adjust force parameters, significantly reducing damage rates in subsequent grasping

First, embodiment: Intelligence originates from physical interaction rather than abstract algorithms. The intelligent behaviors of supply chains do not rely solely on offline reasoning of digital algorithms but stem from direct interactions between physical entities and the environment. For example, the classification decision of intelligent sorting equipment needs to integrate physical contextual information such as product appearance (e.g., packaging color, size) identified by visual sensors, product quality perceived by weight sensors, and real-time speed of the sorting conveyor belt, rather than relying solely on a pre-set product classification database. If the conveyor belt speed decreases due to mechanical failure, the sorting equipment can perceive this change through sensors and automatically adjust the sorting rhythm to avoid cargo congestion; in contrast, traditional sorting systems rely on a fixed sorting rhythm, which leads to cargo congestion or missed sorting once the conveyor belt speed changes.

Second, contextuality: Decisions depend on specific scenarios rather than general solutions. Supply chain decision-making and execution are highly context-dependent, requiring dynamic adjustments based on specific physical situations, and the same decision scheme may produce drastically different results in different scenarios. For example, in order delivery priority decisions: prioritizing nearby orders can shorten delivery time and improve customer satisfaction when logistics is smooth; however, this strategy leads to delivery delays when nearby routes are congested due to severe weather (e.g., heavy rain, snowstorms). In such cases, the strategy needs to be adjusted to prioritize distant unobstructed routes, improving overall efficiency through staggered delivery. This context dependence requires supply chain decisions to be closely integrated with the real-time state of the physical environment.

Third, closed-loop: Continuous optimization through feedback to achieve dynamic evolution. Supply chains continuously optimize perception models and reasoning algorithms through feedback data from physical execution, forming a closed-loop collaboration of ‘perception-reasoning-execution-feedback.’ For example, when a robotic arm damages fragile goods (e.g., glassware, precision instruments) due to excessive grasping force, tactile sensors feed back data on the relationship between force and damage to the reasoning model. The model adjusts grasping force parameters through algorithmic analysis and continuously fine-tunes them based on feedback data in subsequent grasping processes. This closed-loop optimization mechanism enables supply chain embodied intelligence to continuously adapt to changes in the physical environment and improve collaboration accuracy.

In summary, supply chain embodied intelligence breaks through the separation between digital and physical aspects of traditional intelligent supply chains, achieving deep integration of digital technologies and physical supply chains. It endows supply chains with adaptability to dynamic environments, enabling them to real-time perceive changes in physical situations, dynamically generate adaptive decisions, accurately execute physical operations, and continuously optimize through feedback, thus addressing the uncertainty challenges facing global supply chains.

To further clarify the uniqueness of supply chain embodied intelligence, a comparative analysis from core dimensions is presented in Table 2. It clearly outlines the core differential advantages of supply chain embodied intelligence: breaking away from the single logic of digital optimization in traditional intelligent supply chains, taking real-time interactions between physical entities and the environment as the source of intelligence, and realizing dynamic adaptation between decision-making and execution. Compared with the mirror simulation of digital twins and the equipment networking of intelligent IoT, it places greater emphasis on closed-loop collaboration and physical implementation capabilities of ‘perception-reasoning-execution-feedback,’ truly breaking the barrier between digital and physical supply chains and providing more adaptive solutions for dynamic and complex scenarios.

**Table 2:** Comparison of Core Dimensions among Supply Chain Embodied Intelligence, Digital Twin Supply Chains, and Intelligent IoT Supply Chains.

Concept	Perception Method	Decision Logic	Execution Mode	Feedback Mechanism
Supply Chain Embodied Intelligence	Physical interaction-based multimodal real-time perception	Context-adaptive dynamic decision-making	Direct physical mapping of digital decisions	Closed-loop iterative continuous optimization
Digital Twin Supply Chain	Digital model-based simulated perception	Digital mirror-based optimization decision-making	Indirect execution driven by digital instructions	Static feedback for local adjustment
Intelligent IoT Supply Chain	Equipment networking-based data collection	General algorithm-based batch decision-making	Independent equipment operation	Data feedback for algorithm update

### 3.2. Characteristic dimensions

**Fig 1:**
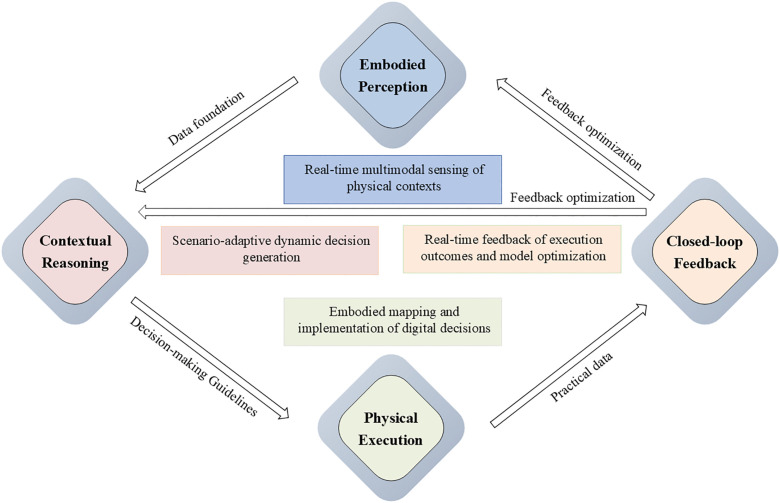
Characteristic Dimensions of Supply Chain Embodied Intelligence.

Based on the core connotation of supply chain embodied intelligence, this study deconstructs it into four characteristic dimensions: embodied perception, contextual reasoning, physical execution, and closed-loop feedback. As shown in Fig 1, these four dimensions are interrelated and mutually supportive: embodied perception provides data foundation for contextual reasoning, contextual reasoning provides decision guidance for physical execution, physical execution provides practical data for closed-loop feedback, and closed-loop feedback in turn feeds back to optimize embodied perception and contextual reasoning, collectively forming a complete characteristic system of supply chain embodied intelligence.

#### 3.2.1. Embodied perception: Multimodal real-time collection of physical situations.

Embodied perception is the foundation of supply chain embodied intelligence, referring to the real-time collection of physical situational data in supply chains through multimodal sensors of physical entities, and it distinguishes itself from the abstract data collection mode of traditional supply chains that relies on manual input or system docking. Its core characteristics can be summarized as physical origin, multimodal type, and real-time collection:

First, the physical origin of data: Data directly stems from physical interactions. Perception data is not abstract statistical data entered manually (e.g., daily sales summaries, monthly inventory reports) but real-time data generated during interactions between physical entities and the environment. For example, real-time position data collected by GPS sensors of logistics vehicles, cargo weight data collected by pressure sensors of warehouse shelves, and storage environment data collected by temperature and humidity sensors of delivery boxes. These data directly reflect the physical state of supply chains, avoiding delays and errors caused by manual input.

Second, the multimodality of data types: Comprehensive coverage of physical situations. Perception data includes multiple types such as visual, tactile, positional, and environmental data, reflecting physical situations of supply chains from different dimensions to ensure data comprehensiveness. Visual data includes product appearance (e.g., packaging damage identification) and shelf layout (e.g., empty space detection); tactile data includes cargo weight (e.g., overload warning) and packaging hardness (e.g., fragile goods identification); positional data includes vehicle coordinates (e.g., delivery trajectory tracking) and robot positioning (e.g., AGV travel path); environmental data includes warehouse temperature and humidity (e.g., fresh produce preservation monitoring) and traffic conditions (e.g., road congestion detection). Multimodal data fusion avoids the limitations of a single data type. For example, visual data alone cannot identify cargo weight, and weight data alone cannot determine whether packaging is damaged.

Third, the real-time nature of data collection: Synchronous with physical events. Sensors operate in conjunction with physical entities, and data collection is almost delay-free with the evolution of physical events, ensuring the timely capture of dynamic changes in physical situations. For example, position data of AGV robots can be updated at high frequency to real-time feedback their travel trajectories and position deviations; speed sensors of sorting conveyor belts can dynamically collect operational data to promptly identify speed anomalies. This real-time nature reserves sufficient time windows for subsequent contextual reasoning and decision adjustments, effectively avoiding decision-making errors caused by data lag.

#### 3.2.2. Contextual reasoning: Dynamic decision generation adapted to scenarios.

Contextual reasoning is the core of supply chain embodied intelligence, referring to intelligent reasoning based on physical situational data from embodied perception combined with specific scenario constraints to generate decision schemes adapted to the current physical situation, rather than the general algorithm optimization results of traditional intelligent supply chains. Its core characteristics can be summarized as contextual basis, dynamic process, and executable results:

First, the contextual basis of reasoning: Close integration with physical constraints. The generation of decision schemes fully considers specific physical scenario constraints, including warehouse space constraints (e.g., shelf height limits, aisle width), logistics time efficiency constraints (e.g., delivery deadlines, order priorities), and product attribute constraints (e.g., shockproof requirements for fragile goods, temperature control requirements for fresh produce). For example, in warehouse cargo placement decisions, the reasoning model needs to integrate the maximum load of shelves (e.g., 500 kg per layer), cargo access frequency (e.g., high-frequency goods need to be easily accessible), and physical attributes of cargo (e.g., heavy goods should be placed on the bottom layer) to generate a placement scheme: high-frequency lightweight goods on the middle layer, low-frequency heavy goods on the bottom layer, and fragile goods in independent areas. In contrast, traditional placement schemes only rely on product categories, failing to consider shelf load and access frequency, which easily leads to shelf overload or low grasping efficiency.

Second, the dynamic nature of the reasoning process: Real-time adjustment with situational changes. When physical situations change, the reasoning model can adjust decision schemes in real-time without manual intervention. For example, in logistics delivery scenarios, if road condition sensors of delivery vehicles detect sudden congestion on the original route (congestion rate increasing from 10% to 70%), the reasoning model immediately calls real-time road condition data of surrounding routes, re-plans delivery paths, selects alternative routes with congestion rates below 15% and fewer than 3 traffic lights, and calculates the delivery time of the new path. If it exceeds the order deadline, it synchronously adjusts the delivery priority of the order and coordinates support from other vehicles. This dynamic adjustment capability ensures that decision schemes are always adapted to the current physical situation.

Third, the executability of reasoning results: Direct connection to physical operations. Decision schemes fully consider the operational capabilities of physical execution subjects, avoiding problems that are digitally feasible but physically unfeasible. For example, grasping decisions generated for robotic arms need to comply with physical parameters such as maximum load (e.g., 50 kg), activity range (e.g., 1.5m radius), and grasping accuracy (e.g., ± 0.1 mm). If the weight of a product exceeds the maximum load of the robotic arm, the reasoning model automatically assigns a heavy-duty robotic arm or adopts a batch grasping strategy instead of generating unexecutable grasping instructions. Traditional reasoning models often ignore the physical capabilities of execution subjects, leading to unimplementable decision schemes. For example, they may generate cargo stacking heights exceeding the lifting height of forklifts, which requires manual readjustment.

#### 3.2.3. Physical execution: Physical implementation of digital decisions.

Physical execution is the guarantee for the practical application of supply chain embodied intelligence, referring to the transformation of digital decisions from the contextual reasoning layer into specific operations of physical supply chains through execution subjects with physical operational capabilities. It realizes direct mapping between digital instructions and physical behaviors, rather than the indirect mode of ‘digital decision-making-manual transmission-physical execution’ in traditional supply chains. Its core characteristics can be summarized as materialized execution subjects, precise execution behaviors, and collaborative execution processes:

First, materialized execution subjects: Possessing physical operational capabilities. Execution subjects are not purely digital systems but equipment or human-machine collaborative units with physical structures and operational functions, including autonomous execution equipment (e.g., AGV robots, industrial robotic arms, intelligent sorters) and human-machine collaborative equipment (e.g., auxiliary handling robotic arms, intelligent delivery vehicles). For example, intelligent sorters achieve product classification through physical structures such as conveyor belts and push plates, and auxiliary sorting robotic arms complete cargo grasping through the movement of mechanical joints. These materialized subjects serve as direct carriers for the implementation of digital decisions, avoiding the limitation that purely digital schemes cannot perform physical operations.

Second, precise execution behaviors: Accurate mapping of digital decisions. Execution subjects can convert specific parameters in digital decisions (e.g., grasping force, travel path, operation timing) into high-precision physical actions, ensuring that execution results are highly consistent with decision objectives. For example, industrial robotic arms precisely regulate joint movement through motors according to force, angle, and other parameters in decision instructions to achieve damage-free cargo handling; AGV robots accurately reach target positions through lidar-based positioning and obstacle avoidance in accordance with planned coordinate paths, with operational accuracy significantly superior to manual operations.

Third, collaborative execution processes: Uninterrupted cross-linkage. Multiple execution subjects can achieve collaborative operations across links and scenarios based on unified digital decisions without manual transfer and connection. For example, after an AGV warehouse robot completes cargo grasping, it synchronizes the ‘cargo in place’ status to sorting robots through the IoT. Sorting robots adjust sorting channels in advance and immediately complete classification upon receiving the cargo. After classification, delivery robots synchronously receive the ‘cargo classified’ signal, pre-plan routes, and accept the cargo. The entire process forms seamless collaboration from warehousing to sorting to delivery, avoiding problems such as waiting, transfer, and connection errors in traditional models.

#### 3.2.4. Closed-loop feedback: Optimized feedback of execution results.

Closed-loop feedback is the evolutionary driving force of supply chain embodied intelligence, referring to the real-time collection of physical execution results through sensors and their feedback to the embodied perception layer and contextual reasoning layer. It continuously optimizes perception models and reasoning algorithms to achieve dynamic iteration of supply chain intelligence, rather than the static mode of fixed and one-time optimization in traditional intelligent supply chains. Its core characteristics can be summarized as real-time feedback data, targeted feedback effects, and cumulative feedback outcomes:

First, real-time feedback data: Synchronous transmission with execution processes. Feedback data is collected in real-time by dedicated sensors deployed on execution subjects, occurring synchronously with physical execution behaviors without manual aggregation or delayed transmission. For example, visual sensors of sorting equipment real-time record the classification results of each product to generate classification accuracy data; GPS and time efficiency sensors of delivery vehicles real-time capture delivery time, road congestion, and other information; tactile sensors of robotic arms real-time feedback grasping success rates and product damage conditions. This data is transmitted to upstream layers within milliseconds through 5G or edge computing technologies, ensuring timely intervention of optimization adjustments.

Second, targeted feedback effects: Accurate positioning of optimization links. Feedback data is not generalized overall results but can accurately correspond to problems in specific links such as perception and reasoning, providing a basis for targeted optimization. For example, if feedback data shows that classification errors are concentrated in products with similar appearances, the optimization direction is clearly the visual recognition algorithm of the perception layer (adding product detail feature extraction); if feedback data indicates that delivery time delays are mainly caused by deviations in congestion time estimation, the path planning parameters of the reasoning layer are directly iterated (adjusting congestion road time coefficients); if feedback data shows a high damage rate of fragile goods grasped by robotic arms, the focus is on optimizing the force control model of the reasoning layer, avoiding efficiency waste caused by generalized adjustments.

Third, cumulative feedback outcomes: Continuous upgrading of intelligence levels. With the continuous accumulation of feedback data, the performance of perception models and reasoning algorithms shows an upward trend of iterative optimization, forming a positive cumulative effect where richer data leads to higher intelligence levels. For example, the grasping model of robotic arms achieves a significant improvement in grasping success rate through continuous iterative optimization of feedback data; the delivery path planning model completes algorithm iteration based on massive road condition feedback information, greatly improving the accuracy of delivery time efficiency. After long-term closed-loop optimization, the overall collaboration efficiency, operational accuracy, and environmental adaptability of supply chains will steadily enhance, gradually forming intelligent operational characteristics of self-learning and self-evolution.

## 4. Theoretical framework of embodied intelligence-driven adaptive collaboration in supply chains

**Fig 2:**
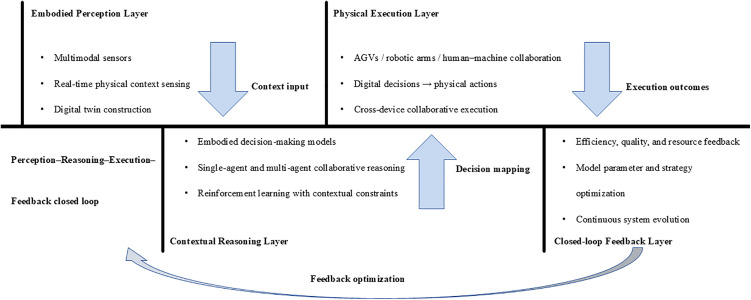
The Four-Dimensional Adaptive Collaboration Theoretical Framework.

Based on the core connotation and characteristic dimensions of supply chain embodied intelligence, this study constructs a four-dimensional adaptive collaboration theoretical framework of ‘embodied perception-contextual reasoning-physical execution-closed-loop feedback.’ As shown in Fig 2, this framework takes embodied cognition theory as the core logic, integrates the subject collaboration idea of complex adaptive system theory and the closed-loop cycle technical path of embodied intelligence theory in robotics, achieves deep coupling between digital technologies and physical supply chains, and promotes the transformation of supply chains from a traditional passive response model to an active perception-dynamic decision-precise execution-continuous optimization embodied adaptive model.

### 4.1. Theoretical integration logic

The three theories form a clear hierarchical support relationship in the framework, with specific logic as follows:

Core logic layer: Embodied cognition theory provides the core logic that intelligence arises from physical interactions, clarifying that supply chain intelligence should originate from dynamic interactions between physical entities and the environment rather than solely relying on abstract reasoning of digital algorithms, providing theoretical basis for the construction of the embodied perception layer and physical execution layer in the framework.

System framework layer: Complex adaptive system theory provides systematic thinking for multi-agent collaborative evolution, guiding the design of interaction mechanisms among various layers and agents (equipment, enterprise nodes, human-machine units) in the framework, providing support for the multi-agent collaboration module of the contextual reasoning layer and the system optimization logic of the closed-loop feedback layer.

Embodied intelligence theory in robotics provides a closed-loop technical path of ‘perception-decision-execution-feedback,’ clarifying the technical implementation methods of each layer and offering technical guarantee for the feasibility of framework implementation.

### 4.2. Functional and interaction logic of each layer

#### 4.2.1. Embodied perception layer: Multimodal fusion perception of physical situations.

The embodied perception layer is the data entry point for adaptive collaboration in supply chains, with the core goal of achieving real-time, comprehensive, and accurate perception of physical supply chain situations, providing high-quality physical situational data for the contextual reasoning layer, and solving the problems of abstract, delayed, and one-sided data in traditional supply chains.

The perception subjects of the embodied perception layer are physical entities deployed in core supply chain links such as warehousing, sorting, and delivery, including multimodal sensors and intelligent equipment (e.g., AGV robots, intelligent shelves). In the warehousing link, shelf pressure sensors, RFID tags, and robots’ visual and lidar sensors work together to perceive cargo weight, position, and environmental obstacles; in the sorting link, visual and weight sensors of conveyor belts cooperate with tactile sensors of robotic arms to complete multi-dimensional product identification; in the delivery link, GPS and road condition sensors of vehicles link with temperature, humidity, and vibration sensors of delivery boxes to ensure the safety and time efficiency of cargo transportation.

Perception data is divided into three categories: spatial, attribute, and environmental, which are processed through spatial coordinate mapping, attribute classification algorithms, and real-time data cleaning algorithms respectively. Multi-sensor fusion technologies such as Kalman filtering and D-S evidence theory are introduced to construct a physical situation digital twin synchronized in real-time with the physical supply chain, achieving low-latency and high-precision data transmission and mapping, and providing reliable and accurate data support for subsequent decision-making.

To formally characterize the embodied perception process, we define the multimodal perception function. Let $d_{visual}(t)$, $d_{tactile}(t)$, and $d_{spatial}(t)$ represent the raw data streams from visual, tactile, and spatial sensors at time t, respectively. The fused physical situational state $S_{perceived}(t)$ is generated through a fusion function $F_{fusion}$:


$$\begin{array}{c} S_{perceived}(t)=F_{fusion}\left(d_{visual}(t),d_{tactile}(t),d_{spatial}(t)\right) \end{array}$$

The fusion function $F_{fusion}$ can be implemented through techniques such as Kalman filtering for spatial-temporal alignment or D-S evidence theory for uncertainty reasoning. This formalization clarifies how heterogeneous, real-time sensory inputs are transformed into a unified state representation that serves as the foundation for subsequent contextual reasoning processes.

#### 4.2.2. Contextual reasoning layer: Embodied intelligent decision-making and collaborative planning.

The contextual reasoning layer is the decision-making hub for adaptive collaboration in supply chains, with the core goal of generating collaborative decision schemes adapted to the current physical situation based on physical situational data from the embodied perception layer and combined with physical constraints of supply chains.

The reasoning model integrates embodied cognition theory and reinforcement learning algorithms. Recent advances in contextual reinforcement learning for supply chain management have demonstrated that agents trained with offline data and adapted online can effectively generalize across diverse operational environments [33], providing a technical foundation for the adaptive decision-making required in our framework. Specifically, the model extracts spatial situational features through convolutional neural networks, analyzes environmental temporal changes through recurrent neural networks, integrates physical constraints such as equipment load and time efficiency requirements, and iteratively generates decision schemes with collaborative efficiency maximization as the reward function. This layer includes single-agent and multi-agent collaborative reasoning modules: the single-agent module generates locally optimized decisions for individual nodes such as warehousing and delivery (e.g., warehouse cargo placement, delivery path planning); the multi-agent module focuses on cross-node collaboration (e.g., global decisions for production-warehousing and warehousing-delivery), ensuring that decisions shift from disembedded to embodied and adapt to physical scenario requirements.

The core parameter design of the reinforcement learning algorithm is as follows: the state space includes physical situational data (e.g., shelf load, road congestion rate), equipment state data (e.g., robotic arm load rate, vehicle endurance), and order demand data (e.g., priority, deadline); the action space includes equipment operation instructions (e.g., grasping force, travel path adjustment) and collaborative strategy instructions (e.g., order priority adjustment, resource scheduling scheme); the reward function comprehensively considers three indicators: collaboration efficiency (e.g., order fulfillment time), operational accuracy (e.g., product damage rate), and resource utilization rate (e.g., equipment idle rate), ensuring the comprehensiveness and adaptability of decision schemes.

To provide a rigorous algorithmic description, the contextual reasoning process is formalized as a reinforcement learning problem defined by the tuple ⟨S,A,P,R,γ⟩:

State space S: Includes physical situational data (e.g., shelf load $L_{shelf}$, road congestion rate $C_{road})$, equipment state data (e.g., robotic arm load rate $U_{arm}$, vehicle endurance $E_{vehicle})$, and order demand data (e.g., priority $P_{order}$, deadline $T_{deadline})$.

Action space A: Includes equipment operation instructions (e.g., grasping force $F_{grasp}$, travel path adjustment ΔPath) and collaborative strategy instructions (e.g., order priority adjustment ΔPriority, resource scheduling scheme Schedule).

Reward function R(s,a): Designed to maximize a weighted combination of three indicators:


$$\begin{array}{c} R(s,a)=w_{\text{1}}\cdot R_{efficiency}+w_{\text{2}}\cdot R_{accuracy}+w_{\text{3}}\cdot R_{utilization} \end{array}$$

where $R_{efficiency}$ reflects order fulfillment time reduction, $R_{accuracy}$ reflects operational error reduction (e.g., product damage rate), and $R_{utilization}$ reflects resource usage optimization (e.g., equipment idle rate), with $w_{\text{1}}+w_{\text{2}}+w_{\text{3}}=\text{1}$.

The optimal policy $\pi^{*}$ is obtained by maximizing the expected cumulative discounted reward:


$$\begin{array}{c} \pi^{*}=\text{arg}{\text{max}}_{\pi }E\left[\sum_{t=\text{0}}^{T}\gamma^{t}R(s_{t},a_{t})|\pi \right] \end{array}$$

This formalization ensures that the reasoning layer generates decisions that are not only context-adaptive but also systematically optimized toward global supply chain performance objectives.

#### 4.2.3. Physical execution layer: Physical mapping and operation of digital decisions.

The physical execution layer is the operational terminal for adaptive collaboration in supply chains, with the core goal of transforming digital decisions from the contextual reasoning layer into specific operations of physical supply chains.

Execution subjects are divided into three categories: autonomous execution equipment (e.g., AGV robots, intelligent sorting systems), human-machine collaborative equipment (e.g., auxiliary sorting robotic arms), and cross-equipment collaborative systems. Relying on digital-physical mapping technology (converting decision parameters into physical control instructions) and real-time communication technology (5G and edge computing ensuring low-latency interaction), accurate implementation of decision instructions is achieved. Execution modes include autonomous execution (e.g., robot autonomous handling), human-machine collaboration (e.g., robotic arm sorting combined with manual exception handling), and cross-equipment collaboration (e.g., seamless connection between warehousing, sorting, and delivery links), effectively eliminating the barrier between digital instructions and physical execution and ensuring efficient collaboration in the execution process.

#### 4.2.4. Closed-loop feedback layer: Real-time feedback and model optimization of execution results.

The closed-loop feedback layer is the evolutionary engine for adaptive collaboration in supply chains, with the core goal of real-time feedback of physical execution results to the perception and reasoning layers to achieve dynamic model optimization.

Feedback data includes three categories: efficiency (e.g., order fulfillment time, equipment operation efficiency), quality (e.g., product damage rate, classification accuracy), and resources (e.g., equipment load rate, energy consumption). After preprocessing by edge computing, it is transmitted to upstream layers through encryption with millisecond-level transmission delay. This layer optimizes the perception model by adjusting sensor deployment density and fusion algorithm weights, and optimizes the reasoning model by adjusting the reinforcement learning reward function and iterating algorithm parameters, forming an adaptive closed loop of ‘perception-reasoning-execution-feedback.’

As shown in Table 3, it clearly presents the corresponding relationship between the core functions, key supporting technologies, and typical application scenarios of each layer (perception-reasoning-execution-feedback), while clarifying the core indicator improvement directions of each layer, making the implementation logic of the theoretical framework more intuitive.

**Table 3:** Correspondence between Functions, Technologies, and Applications of the Four-Dimensional Architecture of Embodied Intelligent Supply Chains.

Architecture Layer	Core Function	Key Supporting Technologies	Core Indicator Improvement Directions	Typical Application Scenarios
Embodied Perception Layer	Multimodal real-time collection of physical situations	Lidar + Visual Camera + Force Sensor + Kalman Filter + D-S Evidence Theory	Perception accuracy rate; Data real-time performance (ms); Multimodal fusion accuracy	Warehouse shelf load monitoring; Delivery road condition capture; Product attribute identification
Contextual Reasoning Layer	Dynamic adaptive decision-making and collaborative planning	Reinforcement Learning + Recurrent Neural Network + Constraint Optimization Algorithm + Convolutional Neural Network	Decision adaptability index; Collaboration efficiency (order fulfillment time); Response speed (decision generation time)	Dynamic replenishment priority adjustment; Congested path re-planning; Sorting strategy adaptation
Physical Execution Layer	Physical implementation of digital decisions and cross-link collaboration	Autonomous Mobile Robots + Robotic Arms + 5G Real-Time Communication + Digital-Physical Mapping	Execution accuracy rate; Operational stability; Cross-equipment collaboration success rate	Seamless connection between warehousing, sorting, and delivery; Precise product grasping; Dynamic obstacle avoidance
Closed-Loop Feedback Layer	Model iteration and intelligent optimization	Edge Computing + Feedback Data Preprocessing + Parameter Tuning Algorithm	Model optimization efficiency (iterations to convergence); Error reduction range; Intelligent evolution speed	Sorting error correction; Path planning model iteration; Grasping force optimization

The core indicator directions proposed in Table 3 align with broader trends in supply chain performance measurement research. Existing studies have established multidimensional frameworks for evaluating supply chain adaptability, including metrics for responsiveness, flexibility, and operational efficiency. The four-layer indicator structure proposed here extends these frameworks by explicitly incorporating embodied physical execution and closed-loop feedback as distinct evaluative dimensions, which have been largely overlooked in traditional supply chain performance measurement. Future empirical work should develop quantitative measures for each indicator direction and validate them through case studies and simulation experiments.

### 4.3. Inter-layer interaction mechanism

Each layer achieves collaborative operation through data flow and instruction transmission, with specific interaction logic as follows:

1. Data upload: The embodied perception layer real-time transmits processed multimodal physical situational data to the contextual reasoning layer, providing data input for decision generation; the physical execution layer real-time transmits state data and result data during execution to the closed-loop feedback layer, providing a basis for model optimization.
2. Instruction download: The contextual reasoning layer accurately transmits generated collaborative decision instructions to the physical execution layer to guide physical operations; the closed-loop feedback layer transmits optimization parameters and adjustment instructions to the embodied perception layer and contextual reasoning layer respectively to drive model iteration.
3. Collaboration trigger: When sudden changes occur in the physical situation (e.g., road congestion, equipment failure), the embodied perception layer real-time captures and triggers cross-layer collaboration. The contextual reasoning layer quickly adjusts decisions, the physical execution layer synchronously responds, and the closed-loop feedback layer immediately optimizes, ensuring the supply chain system quickly adapts to changes.

## 5. Mechanisms of embodied intelligence-driven adaptive collaboration in supply chains

With the capability of the four-dimensional adaptive collaboration theoretical framework of ‘embodied perception-contextual reasoning-physical execution-closed-loop feedback,’ embodied intelligence deeply integrates into various supply chain links. Through multi-dimensional mechanisms, it breaks the information barriers and delayed response pain points of traditional supply chain collaboration, promoting the adaptive upgrading of supply chains from passive adaptation to active response, from local optimization to global collaboration, and from static operation to dynamic evolution. The inherent operational logic of embodied intelligence-driven adaptive collaboration in supply chains is systematically analyzed from three core dimensions: contextual coupling, subject collaboration, and evolutionary optimization, as shown in Table 4.

**Table 4:** Mechanisms of Embodied Intelligence-Driven Adaptive Collaboration in Supply Chains.

Operation Mechanism	Core Logic	Trigger Conditions	Key Operational Logic
Contextual Coupling Mechanism	Real-time matching between physical situations and digital decisions to shorten response cycles	Sudden disruptions (congestion/equipment failure), periodic fluctuations (logistics peaks), cumulative changes (increased shelf load)	Perception layer captures real-time physical state $S_{perceived}(t)$; 2. Compute divergence from modeled state $S_{modeled}(t)\;$; 3. If divergence > threshold τ, trigger reasoning layer to generate new decision $D_{new}(t)$; 4. Execution layer implements $D_{new}(t)$; 5. Feedback layer monitors execution outcomes for further refinement Summary flow: Perception captures → Reasoning adjusts → Execution responds → Feedback optimizes
Subject Collaboration Mechanism	Data sharing and behavioral negotiation among multiple embodied intelligent agents to achieve global collaborative optimization	Cross-link connection needs (warehousing-sorting-delivery linkage), resource scheduling needs (equipment/capacity allocation)	Agents share real-time state data via IoT; 2. Multi-agent MDP formulates collaborative strategies; 3. Each agent executes assigned actions based on negotiated policy; 4. Execution outcomes are fed back to update the joint policy; 5. Negotiation rules iteratively refined through repeated interactions Summary flow: Agents share data→ Collaborative reasoning formulates → Multi-agent synchronous execution → Feedback optimizes negotiation rules
Evolutionary Optimization Mechanism	Continuous iteration of perception models and reasoning algorithms driven by closed-loop feedback to improve system intelligence levels	Data accumulation reaching thresholds, emergence of new scenario needs, unmet execution effects	Collect execution feedback data $D_{feedback}$; 2. Compute loss L(Θ) based on feedback deviation from target; 3. Update parameters $\Theta_{new}=\Theta_{old}-\eta \nabla_{\Theta }L$; 4. Verify optimization effects through scenario testing; 5. If verified, solidify into standard strategy; otherwise, continue iteration Summary flow: Collect feedback → Iteratively optimize → Verify effects → Solidify strategies → Adapt to new scenarios

### 5.1. Contextual coupling mechanism: Real-time matching between physical situations and digital decisions

The contextual coupling mechanism is triggered by three types of situational changes: sudden (e.g., road congestion), periodic (e.g., logistics peaks), and cumulative (e.g., increased shelf load), which drives the rapid operation of the closed-loop process of ‘perception-decision-execution-feedback.’ Taking the logistics congestion scenario as an example: the perception layer real-time captures congestion data (congestion rate, estimated travel time) through road condition sensors of delivery vehicles and synchronously transmits it to the contextual reasoning layer; the reasoning layer immediately calls real-time data of surrounding routes, dynamically adjusts path planning schemes and order delivery priorities combined with constraints such as order deadlines and vehicle endurance; the execution layer’s delivery vehicles travel according to the new path, while warehouse robots adjust the sorting order based on the updated delivery priorities; the feedback layer real-time collects travel efficiency and order fulfillment progress data of the new path, and immediately feeds back to the reasoning layer for further optimization if delays are still predicted. This process significantly shortens the matching cycle between decisions and dynamic situations, with response efficiency significantly superior to traditional manual decision-making models.

To provide a rigorous characterization of how physical situations and digital decisions are coupled, we formalize the mechanism as follows.

Let $S_{perceived}(t)$ denote the real-time physical state perceived by the embodied perception layer at time t, and let $S_{modeled}(t)$ denote the state assumed by the existing digital model. The set of physical constraints (e.g., shelf load limits, delivery deadlines, equipment specifications) is denoted by C.

Definition 1 (Coupling Function). The generation of a new decision $D_{new}(t)$ in response to a perceived physical state is defined by the coupling function:


$$\begin{array}{c} D_{new}(t)=f_{coupling}\left(S_{perceived}(t),C\right) \end{array}$$

where $f_{coupling}$ integrates the current physical state with operational constraints to produce a context-adaptive decision.

Definition 2 (Triggering Condition). The re-coupling between the physical situation and digital decision is triggered when the divergence between the perceived state and the modeled state exceeds a predefined threshold τ:

$Triggerre-couplingif:\;Div\left(S_{perceived}(t),S_{modeled}(t)\right)>\tau$

where Div(·,·) is a suitable divergence measure (e.g., weighted Euclidean distance over relevant state dimensions such as congestion rate, equipment load, and order priority).

This formalization captures the essential property that embodied intelligence does not operate on a fixed schedule but is event-driven, responding to meaningful discrepancies between the physical world and its digital representation. Compared to traditional models where decisions are updated based on fixed periodic cycles, this mechanism enables near-instantaneous adaptation with significantly shorter response times.

### 5.2. Subject collaboration mechanism: Interaction and collaboration among multiple embodied intelligent agents

The subject collaboration mechanism realizes global optimization goals based on a closed-loop model of ‘data sharing-behavioral negotiation-collaborative execution-result feedback.’ Taking the e-commerce peak scenario as an example: embodied intelligent agents in various links such as warehousing, sorting, and delivery (intelligent shelves, sorting robots, delivery vehicles) achieve real-time data sharing through the IoT, including order volume, equipment load, inventory status, and road condition information; the multi-agent collaborative reasoning module formulates collaborative operation strategies based on shared data, such as adjusting sorter operation speed according to order peaks, allocating warehouse shipment volume according to delivery vehicle capacity, and optimizing shipment timing according to road condition information; during execution, each agent synchronously updates its operational status in real-time. If equipment failure or order backlog occurs in a certain link, collaborative adjustments are immediately triggered. For example, when a sorter fails, the system automatically allocates backup equipment, and warehouse robots temporarily suspend cargo delivery to this link to avoid accumulation. the feedback layer feeds back execution results of each link (sorting accuracy, delivery time efficiency) to the collaborative reasoning module, continuously optimizing negotiation rules and collaborative strategies, and effectively avoiding disconnection between links in traditional supply chain models.

The multi-agent collaborative process is formalized using a multi-agent Markov Decision Process (MDP) framework to capture the dynamic interactions and negotiations among embodied intelligent agents.

Definition 3 (Multi-Agent Collaborative MDP). The collaboration among N embodied intelligent agents is defined by the tuple:

$\langle N,S,\{A_{i}\overset{N}{\underset{i=\text{1}}{\}}},P,\{R_{i}\overset{N}{\underset{i=\text{1}}{\}}},\gamma \rangle$

where:

N  is the number of agents (e.g., warehouse robots, sorting robots, delivery vehicles);

S is the joint state space representing the combined physical and operational states of all agents and the environment;

$A_{i}$ is the action space of agent i, including its available operations (e.g., path selection, grasping force, sorting speed);

$P:S\times A_{\text{1}}\times \cdots \times A_{N}\to \Delta (S)$ is the state transition probability function;

$R_{i}:S\times A_{\text{1}}\times \cdots \times A_{N}\to R$ is the reward function for agent i;

γ∈[0,1) is the discount factor.

The collaborative objective is to find a joint policy $\pi =(\pi_{\text{1}},\ldots ,\pi_{N})$ that maximizes the global welfare:

$\pi^{*}=\text{arg}\underset{\pi }{\text{max}}E\left[\sum_{t=\text{0}}^{T}\sum_{i=\text{1}}^{N}\gamma^{t}R_{i}(s_{t},a_{t})|\pi \right]$

This formalization reveals how embodied intelligence fundamentally changes collaborative logic: instead of following pre-programmed sequential workflows, agents engage in dynamic, data-driven negotiation where each agent’s actions are continuously adjusted based on the real-time states and behaviors of other agents. The emergence of globally optimized collaborative behavior arises from this ongoing process of shared perception, behavioral negotiation, and joint policy refinement.

### 5.3. Evolutionary optimization mechanism: Continuous intelligence upgrading driven by closed-loop feedback

The evolutionary optimization mechanism follows a closed-loop path of ‘data accumulation-model iteration-effect verification-strategy solidification,’ realizing autonomous learning and capability improvement of intelligent systems relying on dynamic feedback mechanisms. Taking the intelligent sorting system as an example: in the initial operation stage, the system completes core function implementation through basic algorithm and process construction, with a classification accuracy of approximately 92% and a high manual intervention rate; as practical application scenarios progress, the system continuously collects feedback data, including classification error cases (e.g., misclassification of products with similar appearances) and equipment operation parameter deviations (e.g., sorting delays caused by conveyor belt speed fluctuations); based on this data, the visual recognition algorithm of the perception layer (adding product detail feature extraction dimensions) and the classification decision model of the reasoning layer (adjusting classification thresholds and priority weights) are optimized; the optimization effect is verified through multiple rounds of scenario testing. If the classification accuracy increases to over 98% and the manual intervention rate drops below 5%, the optimized algorithms and parameters are solidified into standard operating strategies; subsequently, as new product types and new scenario needs emerge, the above closed-loop process is repeated to achieve continuous upgrading of system intelligence. Ultimately, the classification accuracy and operational stability of the system are significantly improved, while promoting phased breakthroughs in the collaborative response efficiency of various supply chain links.

The closed-loop evolutionary optimization is formalized as an iterative parameter optimization process driven by execution feedback.

Definition 4 (Feedback-Driven Parameter Optimization). Let $\Theta =(\Theta_{P},\Theta_{D})$ denote the combined parameter set, where $\Theta_{P}$ represents the parameters of the perception model (e.g., sensor fusion weights, feature extraction parameters) and $\Theta_{D}$ represents the parameters of the decision model (e.g., policy network weights, reward function coefficients). The loss function at iteration k based on execution feedback data $\overset{\left(k\right)}{\underset{feedback}{D}}$ is defined as:

$L(\Theta^{\left(k\right)};\overset{\left(k\right)}{\underset{feedback}{D}})=\alpha \cdot L_{efficiency}+\beta \cdot L_{accuracy}+\delta \cdot L_{stability}$

where $L_{efficiency}$ measures deviation from target operational efficiency, $L_{accuracy}$ measures errors in execution precision, $L_{stability}$ measures system stability fluctuations, and α,β,δ are weighting coefficients with α+β+δ=1.

The parameter update rule follows gradient-based optimization:

$\Theta^{\left(k+\text{1}\right)}=\Theta^{\left(k\right)}-\eta \nabla_{\Theta }L(\Theta^{\left(k\right)};\overset{\left(k\right)}{\underset{feedback}{D}})$

where η is the learning rate.

This formalization captures the self-improving nature of embodied intelligent supply chains. As execution data accumulates with operational time, the iterative parameter updates lead to progressively improved performance. The convergence property:


$$\begin{array}{c} \underset{k\to \infty }{\text{lim}}L(\Theta^{\left(k\right)})\to L_{min} \end{array}$$

represents the system’s capacity for continuous intelligence evolution, where the gap between actual and optimal performance narrows over time. Compared to traditional static optimization models where parameters are set once and remain fixed, this mechanism enables the supply chain to autonomously adapt to emerging patterns, new product types, and shifting environmental conditions without manual reconfiguration.

## 6. Case study

### 6.1. Pharmaceutical warehousing scenario: Practical application of humanoid robots in full-process flexible operations

#### 6.1.1. Cooperation background and scenario needs.

Pharmaceutical warehousing operations have extremely high requirements for operational accuracy and process standardization. Traditional automated equipment has obvious limitations: facing different shapes and specifications of drug box SKUs, mechanical parameters need to be re-tuned, resulting in high adaptation costs; the code scanning and warehousing link relies on fixed placement angles and cannot independently adjust material postures, easily leading to process bottlenecks. Star Dynamic Era [34] launched an embodied intelligent logistics solution [17], which was practically applied in real pharmaceutical warehousing scenarios.

#### 6.1.2. Core implementation technologies of embodied intelligence.

Embodied Perception Layer: Adopts a multimodal scheme of ‘vision + touch + spatial perception.’ The humanoid robot ‘Star Dynamic L7’ is equipped with a self-developed five-finger dexterous hand, capturing the shape and placement angle of drug boxes through a wrist cross-axis structure, and real-time identifying barcode positions through visual sensors to comprehensively perceive operational objects and environmental information.

Contextual Reasoning Layer: Relies on an end-to-end VLA embodied model to realize a real-time closed loop of ‘visual recognition-action planning-task execution.’ When the position of a drug box is artificially adjusted, the model can quickly re-plan the grasping path and independently adjust the flipping angle of the drug box until the barcode is aligned with the scanning area.

Physical Execution Layer: The robot completes full-process operations according to reasoning instructions, flexibly grasping drug boxes of different specifications from material boxes, accurately aligning with scanning equipment for warehousing entry, and then stably placing them on the feeding conveyor belt. The entire set of actions is completed without manual intervention, adapting to non-dead-angle grasping of multiple drug box categories.

Closed-Loop Feedback Layer: Grasping data and adaptation parameters generated during operations feed back to the model, continuously optimizing the operational accuracy and posture adjustment logic of the robotic arm, forming an evolutionary closed loop of ‘practice-optimization-iteration.’

#### 6.1.3. Implementation value and industrial significance.

This solution fills the technical gap in flexible pharmaceutical warehousing operations. Compared with traditional automated equipment, it does not require parameter re-tuning when replacing SKUs, significantly shortening adaptation time and improving the efficiency of multi-category operations. Its core value lies in realizing the transformation from ‘fixed program-driven’ to ‘scenario dynamic adaptation,’ providing a reusable embodied intelligence implementation paradigm for multi-SKU and high-frequency switching scenarios such as pharmaceuticals and e-commerce.

## 7. Future research directions

### 7.1. Theoretical expansion

1. Construct a measurement index system for supply chain embodied intelligence, drawing upon established multidimensional performance measurement frameworks in the supply chain literature. Future work should clarify the quantification methods of core indicators such as perception accuracy, reasoning adaptability, execution collaboration degree, and feedback optimization efficiency, and design a scientifically rigorous indicator weight allocation model validated through empirical studies.
2. Conduct in-depth research on the inherent mechanism of human-machine collaborative embodied decision-making, clarify the decision-making boundaries and responsibility division between humans and embodied intelligent equipment in different scenarios (e.g., high-risk operations, complex decision-making scenarios), and explore the influencing factors and implementation paths of human-machine trust establishment.
3. Explore the value creation path of embodied intelligence-driven supply chains, analyze the value formation mechanism in terms of efficiency improvement, innovation empowerment, and ecosystem construction, and construct a multi-dimensional value evaluation framework.

### 7.2. Technology integration

1. Develop anti-interference fusion technology for multimodal perception data to address problems such as data heterogeneity, noise interference, and poor synchronization in complex supply chain environments (e.g., warehouse dust, logistics vibration), improving the reliability and accuracy of perception data.
2. Explore the deep integration path of generative AI and embodied reasoning, leveraging the innovative capabilities of generative AI such as large language models to assist contextual reasoning models in generating diverse decision schemes. Recent studies explore the integration of generative AI (e.g., large language models) and embodied reasoning to generate diverse decision schemes. Research on large language models in blockchain-based supply chain finance [22] shows their integration with blockchain enhances financing efficiency and provides reliable decision support. Similarly, exploring how generative AI can augment embodied reasoning for complex unknown scenarios represents a promising research frontier for enhancing adaptability and innovative decision-making capabilities.
3. Study the combination mode of blockchain and embodied intelligence, realizing trusted data sharing, automatic execution of smart contracts, and responsibility traceability in supply chains through blockchain technology, addressing trust issues and risk management challenges in multi-agent collaboration.

### 7.3. Practical implementation

1. Formulate differentiated embodied intelligence implementation paths for the supply chain characteristics of different industries such as manufacturing, retail, and fresh produce logistics, clarifying the core application scenarios, key technical configurations, and implementation priorities of each industry.
2. Develop low-cost embodied intelligence solutions for small and medium-sized enterprises, including modular software systems, economical hardware equipment, and third-party technical service models, reducing the technical application threshold for small and medium-sized enterprises.
3. Study risk governance strategies for embodied intelligent supply chains, establishing risk identification, assessment, and prevention systems for potential risks such as algorithmic bias, equipment failure, data security, and privacy leakage, ensuring the stable operation of supply chains.

## 8. Conclusion

This study introduces embodied intelligence theory into the field of supply chain management, proposing for the first time the core concept and connotation of supply chain embodied intelligence, clarifying its three core characteristics (embodiment, contextuality, closed-loop) and four characteristic dimensions (embodied perception, contextual reasoning, physical execution, closed-loop feedback), constructing a four-dimensional adaptive collaboration theoretical framework of ‘embodied perception-contextual reasoning-physical execution-closed-loop feedback,’ and revealing three core mechanisms: contextual coupling, subject collaboration, and evolutionary optimization. This study achieves the deep coupling of embodied intelligence and supply chain collaboration, breaks the dual separation between digital technologies and physical supply chains, and promotes the transformation of supply chain management from the traditional data-driven digital optimization paradigm to the physical-digital deep integration embodied adaptive paradigm.

Case studies show that embodied intelligence can effectively enhance the dynamic response capability, collaboration efficiency, and operational accuracy of supply chains, providing a feasible path for the intelligent upgrading of scenarios such as logistics sorting, intelligent warehousing, and flexible delivery. In the future, the academic community needs to further deepen cross-disciplinary research between embodied intelligence and supply chain management, improving the theoretical system and measurement methods; enterprises should actively explore the practical application of embodied intelligence technologies, formulating differentiated implementation strategies combined with industry characteristics and their own needs; subsequent research also needs to verify through more empirical data and cases, continuously optimizing the theoretical framework and technical solutions, and providing stronger theoretical support and practical guidance for the development of next-generation intelligent supply chains.

**Large Language Model Usage Statement**: This paper has utilized large language models for translation and polishing. The relevant content has undergone manual verification to ensure the accuracy of the core meaning.
