# Supplementary material for: Embodied intelligence-driven adaptive collaboration in supply chains: A four-dimensional synergy framework and mechanism analysis
Source: PLoS One. 2026 Jun 9;21(6):e0351058. doi: 10.1371/journal.pone.0351058 (PMC13249223; doi:10.1371/journal.pone.0351058)
Supplement: Attachment — Submitted filename: Response to Reviewers.docx [file pone.0351058.s002.docx]

**Response to Reviewers**

Manuscript ID: PONE-D-26-10513

Title: Embodied intelligence-driven adaptive collaboration in supply chains: A four-dimensional synergy framework and mechanism analysis

Journal: PLOS ONE

Dear Dr. Yang (Jack) Lu,

We sincerely thank you and the two reviewers for your careful reading of our manuscript and for providing such constructive and detailed feedback. These comments have been invaluable in strengthening the rigor, clarity, and depth of our work. We have carefully revised the manuscript to address every point raised. Below, we provide a detailed, point-by-point response. All changes in the manuscript are highlighted in the “Revised Manuscript with Track Changes”s file.

Sincerely,

Huiying Xu

(on behalf of all authors)

**Response to Journal Requirements**

1. Please ensure that your manuscript meets PLOS ONE's style requirements, including those for file naming...

**Response**: We have carefully reformatted the entire manuscript according to the PLOS ONE style templates provided. This includes adjustments to the title, author, affiliations, section headings, citation format, reference list style, and file naming conventions. The revised manuscript now fully complies with all journal style requirements.

2. Please note that PLOS One has specific guidelines on code sharing for submissions in which author-generated code underpins the findings in the manuscript. In these cases, we expect all author-generated code to be made available without restrictions upon publication of the work...

**Response**: We fully understand the code sharing policy. Our manuscript is a theoretical study that proposes a conceptual framework and analyzes its mechanisms. No author-generated code was used to produce the findings. Should any code be developed in future follow-up empirical work, we will of course share it in accordance with PLOS ONE’s policy.

3. Please state what role the funders took in the study.

**Response**: We have updated the funding statement. The amended Role of Funder statement is: “The funders had no role in study design, data collection and analysis, decision to publish, or preparation of the manuscript.” Please amend the online submission form on our behalf.

4. Please remove any funding-related text from the manuscript. Funding information should not appear in any section or other areas of your manuscript. We will only publish funding information present in the Funding Statement section of the online submission form.

**Response**: We have completely removed all funding-related text and acknowledgements from the manuscript file. Funding information will now only appear in the Funding Statement section of the online submission form, as requested.

5. Please confirm at this time whether or not your submission contains all raw data required to replicate the results of your study...

**Response**: We confirm that our submission contains all data required to replicate the study findings. As this is a theoretical paper, the “minimal data set” consists of the conceptual arguments, comparative tables, and the case description presented within the manuscript. All such data are contained within the manuscript itself. No external datasets or raw data files were generated or analyzed. Should the editor require any further information, we are happy to provide it.

6. Please update the spelling of the author's name Huiyin Xu/Huiying Xu in the manuscript and in the manuscript submission data (via Edit Submission).

**Response**: We apologize for the inconsistency. We have now unified the spelling as Huiying Xu both in the manuscript and in the submission system, as requested.

**Response to Reviewer #1**

**Comment**: The manuscript would benefit significantly from the inclusion of additional statistical analysis and well-defined mathematical equations to enhance its academic standardization. Incorporating appropriate statistical tools—such as descriptive statistics, correlation analysis, regression models, hypothesis testing or optimization techniques—would strengthen the reliability and validity of the research findings.

**Response**: We are very grateful for this crucial suggestion. We fully agree that our theoretical framework needs a higher degree of formalization to meet academic standards of rigor. While the nature of our study (a theoretical framework proposal) does not involve empirical data for statistical analysis, we have substantially enhanced the manuscript by incorporating well-defined mathematical formulations and formal descriptions of our core mechanisms and architecture. The specific additions are as follows:

1. Formalization of the Embodied Perception Layer (Section 4.2.1): We have added a mathematical definition for multimodal embodied perception to characterize how raw sensor data is fused into a unified physical state. The basic principle is formalized as:

$$S_{perceived}(t)={\mathcal{\mathcal{F}}}_{fusion}\left( d_{visual}(t),d_{tactile}(t),d_{spatial}(t) \right)$$

This clarifies the transformation from raw, heterogeneous data streams to a coherent perceptual input.

2. Formalization of the Contextual Reasoning Layer (Section 4.2.2): We have detailed the reinforcement learning algorithm flow by defining the state space $\mathcal{S}$, action space $\mathcal{A}$, and reward function $R(s,a)$. The decision generation is now described as a policy optimization problem:

$${\pi^{*}}^{=}\arg\max_{\pi}\mathbb{E}\left[ \sum_{t=0}^{T} \gamma^{t}R(s_{t},a_{t}) \right]$$

This provides a rigorous algorithmic backbone for the decision-making module.

3. Formalization of the Three Core Mechanisms (Section 5): We have introduced mathematical descriptions for each mechanism to replace purely macroscopic descriptions:

Contextual Coupling Mechanism (Section 5.1): We define a coupling function $D_{new}(t)=f_{coupling}(S_{perceived}(t),C)$ and a triggering condition $Divergence(S_{perceived}(t),S_{modeled}(t))>\tau$. This formally captures how a discrepancy between the physical world and the digital model triggers adaptive re-decision.

Subject Collaboration Mechanism (Section 5.2): We model multi-agent collaboration using a multi-agent Markov Decision Process (MDP) with the tuple $\langle N,\mathcal{S},\{\mathcal{A}_{i}{\}}_{i=1}^{N},\mathcal{P},\{{\mathcal{\mathcal{R}}}_{i}{\}}_{i=1}^{N},\gamma\rangle$. This mathematically characterizes how multiple embodied agents interact, share data, and negotiate to achieve global optimization.

Evolutionary Optimization Mechanism (Section 5.3): We formulated the closed-loop feedback as an iterative parameter optimization problem, where a loss function $\mathcal{\mathcal{L}}(\Theta)$ driven by execution feedback continuously updates perception and decision parameters $\Theta$.

We believe that the addition of these mathematical formulations and algorithm descriptions significantly elevates the academic standardization of the manuscript, making the proposed framework and mechanisms both rigorous and transparent.

**The full content is as follows:**

To formally characterize the embodied perception process, we define the multimodal perception function. Let $d_{visual}(t)$, $d_{tactile}(t)$, and $d_{spatial}(t)$ represent the raw data streams from visual, tactile, and spatial sensors at time $t$, respectively. The fused physical situational state $S_{perceived}(t)$ is generated through a fusion function ${\mathcal{\mathcal{F}}}_{fusion}$:

$$S_{perceived}(t)={\mathcal{\mathcal{F}}}_{fusion}\left( d_{visual}(t),d_{tactile}(t),d_{spatial}(t) \right)$$

The fusion function ${\mathcal{\mathcal{F}}}_{fusion}$ can be implemented through techniques such as Kalman filtering for spatial-temporal alignment or D-S evidence theory for uncertainty reasoning. This formalization clarifies how heterogeneous, real-time sensory inputs are transformed into a unified state representation that serves as the foundation for subsequent contextual reasoning processes.

To provide a rigorous algorithmic description, the contextual reasoning process is formalized as a reinforcement learning problem defined by the tuple $\langle\mathcal{S},\mathcal{A},\mathcal{P},\mathcal{\mathcal{R}},\gamma\rangle$:

State space $\mathcal{S}$: Includes physical situational data (e.g., shelf load $L_{shelf}$, road congestion rate $C_{road})$, equipment state data (e.g., robotic arm load rate $U_{arm}$, vehicle endurance $E_{vehicle})$, and order demand data (e.g., priority $P_{order}$, deadline $T_{deadline})$.

Action space $\mathcal{A}$: Includes equipment operation instructions (e.g., grasping force $F_{grasp}$, travel path adjustment $\Delta Path)$ and collaborative strategy instructions (e.g., order priority adjustment $\Delta Priority$, resource scheduling scheme $Schedule)$.

Reward function $\mathcal{\mathcal{R}}(s,a)$: Designed to maximize a weighted combination of three indicators:

$$\mathcal{\mathcal{R}}(s,a)=w_{1}\cdot R_{efficiency}+w_{2}\cdot R_{accuracy}+w_{3}\cdot R_{utilization}$$

where $R_{efficiency}$ reflects order fulfillment time reduction, $R_{accuracy}$ reflects operational error reduction (e.g., product damage rate), and $R_{utilization}$ reflects resource usage optimization (e.g., equipment idle rate), with $w_{1}+w_{2}+w_{3}=1$.

The optimal policy $\pi^{*}$ is obtained by maximizing the expected cumulative discounted reward:

$$\pi^{*}=\arg\max_{\pi}\mathbb{E}\left[ \sum_{t=0}^{T} \gamma^{t}\mathcal{\mathcal{R}}(s_{t},a_{t})\mid\pi\right]$$

This formalization ensures that the reasoning layer generates decisions that are not only context-adaptive but also systematically optimized toward global supply chain performance objectives.

To provide a rigorous characterization of how physical situations and digital decisions are coupled, we formalize the mechanism as follows.

Let $S_{perceived}(t)$ denote the real-time physical state perceived by the embodied perception layer at time $t$, and let $S_{modeled}(t)$ denote the state assumed by the existing digital model. The set of physical constraints (e.g., shelf load limits, delivery deadlines, equipment specifications) is denoted by $\mathcal{C}$.

Definition 1 (Coupling Function). The generation of a new decision $D_{new}(t)$ in response to a perceived physical state is defined by the coupling function:

$$D_{new}(t)=f_{coupling}\left( S_{perceived}(t),\mathcal{C} \right)$$

where $f_{coupling}$ integrates the current physical state with operational constraints to produce a context-adaptive decision.

Definition 2 (Triggering Condition). The re-coupling between the physical situation and digital decision is triggered when the divergence between the perceived state and the modeled state exceeds a predefined threshold $\tau$:

$$Triggerre-couplingif:\quad Div\left( S_{perceived}(t),S_{modeled}(t) \right)>\tau$$

where $Div(\cdot,\cdot)$ is a suitable divergence measure (e.g., weighted Euclidean distance over relevant state dimensions such as congestion rate, equipment load, and order priority).

This formalization captures the essential property that embodied intelligence does not operate on a fixed schedule but is event-driven, responding to meaningful discrepancies between the physical world and its digital representation. Compared to traditional models where decisions are updated based on fixed periodic cycles, this mechanism enables near-instantaneous adaptation with significantly shorter response times.

The multi-agent collaborative process is formalized using a multi-agent Markov Decision Process (MDP) framework to capture the dynamic interactions and negotiations among embodied intelligent agents.

Definition 3 (Multi-Agent Collaborative MDP). The collaboration among $N$ embodied intelligent agents is defined by the tuple:

$$\langle N,\mathcal{S},\{\mathcal{A}_{i}{\}}_{i=1}^{N},\mathcal{P},\{{\mathcal{\mathcal{R}}}_{i}{\}}_{i=1}^{N},\gamma\rangle$$

where:

$N$is the number of agents (e.g., warehouse robots, sorting robots, delivery vehicles);

$\mathcal{S}$ is the joint state space representing the combined physical and operational states of all agents and the environment;

$\mathcal{A}_{i}$ is the action space of agent $i$, including its available operations (e.g., path selection, grasping force, sorting speed);

$\mathcal{P}:\mathcal{S}\times\mathcal{A}_{1}\times\cdots\times\mathcal{A}_{N}\to\Delta(\mathcal{S})$ is the state transition probability function;

${\mathcal{\mathcal{R}}}_{i}:\mathcal{S}\times\mathcal{A}_{1}\times\cdots\times\mathcal{A}_{N}\to\mathbb{\mathbb{R}}$ is the reward function for agent $i$;

$\gamma\in[0,1)$ is the discount factor.

The collaborative objective is to find a joint policy $\boldsymbol{\pi}=(\pi_{1},\ldots,\pi_{N})$ that maximizes the global welfare:

$$\boldsymbol{\pi}^{*}=\arg\max_{\boldsymbol{\pi}} \mathbb{E}\left[ \sum_{t=0}^{T} \sum_{i=1}^{N} \gamma^{t}{\mathcal{\mathcal{R}}}_{i}(s_{t},\boldsymbol{a}_{t})\mid\boldsymbol{\pi} \right]$$

This formalization reveals how embodied intelligence fundamentally changes collaborative logic: instead of following pre-programmed sequential workflows, agents engage in dynamic, data-driven negotiation where each agent's actions are continuously adjusted based on the real-time states and behaviors of other agents. The emergence of globally optimized collaborative behavior arises from this ongoing process of shared perception, behavioral negotiation, and joint policy refinement.

The closed-loop evolutionary optimization is formalized as an iterative parameter optimization process driven by execution feedback.

Definition 4 (Feedback-Driven Parameter Optimization). Let $\Theta=(\Theta_{P},\Theta_{D})$ denote the combined parameter set, where $\Theta_{P}$ represents the parameters of the perception model (e.g., sensor fusion weights, feature extraction parameters) and $\Theta_{D}$ represents the parameters of the decision model (e.g., policy network weights, reward function coefficients). The loss function at iteration $k$ based on execution feedback data $D_{feedback}^{(k)}$ is defined as:

$$\mathcal{\mathcal{L}}(\Theta^{(k)};D_{feedback}^{(k)})=\alpha\cdot{\mathcal{\mathcal{L}}}_{efficiency}+\beta\cdot{\mathcal{\mathcal{L}}}_{accuracy}+\delta\cdot{\mathcal{\mathcal{L}}}_{stability}$$

where ${\mathcal{\mathcal{L}}}_{efficiency}$ measures deviation from target operational efficiency, ${\mathcal{\mathcal{L}}}_{accuracy}$ measures errors in execution precision, ${\mathcal{\mathcal{L}}}_{stability}$ measures system stability fluctuations, and $\alpha,\beta,\delta$ are weighting coefficients with $\alpha+\beta+\delta=1$.

The parameter update rule follows gradient-based optimization:

$$\Theta^{(k+1)}=\Theta^{(k)}-\eta\nabla_{\Theta}\mathcal{\mathcal{L}}(\Theta^{(k)};D_{feedback}^{(k)})$$

where $\eta$ is the learning rate.

This formalization captures the self-improving nature of embodied intelligent supply chains. As execution data accumulates with operational time, the iterative parameter updates lead to progressively improved performance. The convergence property:

$$\lim_{k\to\infty} \mathcal{\mathcal{L}}(\Theta^{(k)})\to{\mathcal{\mathcal{L}}}_{min}$$

represents the system's capacity for continuous intelligence evolution, where the gap between actual and optimal performance narrows over time. Compared to traditional static optimization models where parameters are set once and remain fixed, this mechanism enables the supply chain to autonomously adapt to emerging patterns, new product types, and shifting environmental conditions without manual reconfiguration.

**Response to Reviewer #2**

**Comment 1**: The theoretical and practical contributions are too general, lacking a clear comparison with existing literature to explain what this paper truly advances.

**Response**: We fully agree with this critique. We have rewritten the contribution sections (Section 1.2) to ensure every stated contribution is directly contextualized against existing research. Specifically:

In the theoretical contributions (Section 1.2.1), we now explicitly contrast our work with prior literature. For example, we state that while existing research adopts a “disembedded” model that abstracts supply chains into data for algorithm optimization [14-16], our work is the first to treat physical interaction as the source of intelligence. We also clarify that unlike studies focusing solely on digital mirroring (digital twins) or data sharing, our framework creates a closed-loop where physical execution feedback dynamically drives model evolution.

In the practical contributions (Section 1.2.2), we have specified how our framework provides an actionable guide that is distinct from current local optimization practices, helping enterprises shift from isolated link optimization to end-to-end embodied collaboration.

**The full content is as follows:**

First, in contrast to existing literature that treats artificial intelligence as an abstract optimization tool applied to supply chain data models [14-16], this study is among the first to systematically introduce embodied intelligence theory into the field of supply chain management. By clarifying the core concept, connotative boundaries, and characteristic dimensions of supply chain embodied intelligence, it fills the theoretical gap in intelligent supply chain collaboration for physical-digital integrated scenarios. This fundamentally shifts the research bias from a digital-above-all approach to a balanced physical-digital fusion perspective. Second, it breaks through the one-way, data-driven paradigm of traditional intelligent supply chains. While prior research has focused on information sharing or digital twin-based mirror simulation, this study constructs a bi-directional adaptive collaboration mechanism of ‘embodied perception-contextual reasoning-physical execution-closed-loop feedback’. This mechanism integrates the agent co-evolution logic of complex adaptive system theory with the contextual interaction logic of embodied cognition theory into supply chain management, crucially repositioning physical execution not merely as an output but as a generative source of intelligence that dynamically feeds back into decision optimization. This enriches the application scenarios and theoretical depth of complex adaptive system theory in the supply chain field. Third, by deconstructing the inherent logic of embodied intelligence-driven supply chain collaboration, this study identifies three core mechanisms including contextual coupling, subject collaboration, and evolutionary optimization, and further provides mathematical formalizations for each mechanism. It lays a solid theoretical foundation for the paradigm transformation of supply chain management from digital optimization to embodied adaptability, and advances the development of supply chain management theory toward in-depth physical-digital integration. The formal definitions and mathematical descriptions of the above mechanisms also support rigorous theoretical verification and facilitate subsequent empirical research.

The practical value of this study focuses on providing actionable, structured guidance for enterprises to build next-generation intelligent supply chains, distinctly different from current piecemeal technology adoption approaches:

First, it helps enterprises break the persistent separation between digital technologies and physical operations. Rather than deploying technologies in isolation, this study provides an integrated framework for realizing contextual perception and physical execution through the systematic deployment of embodied intelligent equipment (e.g., multimodal sensors, autonomous mobile robots) and the construction of a four-layer collaborative architecture with clearly defined inter-layer interaction protocols.

Second, it guides enterprises to shift from a local mindset of optimizing individual links through algorithms to a systematic mindset of end-to-end embodied collaboration. By specifying the indicator system and implementation path for each architectural layer, the framework enables enterprises to measure and enhance supply chain adaptability to dynamic environments in a structured manner.

Third, it provides specific, theoretically grounded technical paths for the intelligent upgrading of physical supply chain scenarios such as logistics sorting, intelligent warehousing, and flexible production, with each path mapped to corresponding technologies and performance indicators.

**Comment 2**: Existing mechanism descriptions are too macroscopic, failing to reveal how intelligence fundamentally changes the collaborative logic.

**Response**: Thank you for this insightful comment. We have thoroughly revised Section 5 to make the mechanisms more granular. Our revisions include:

Addition of a “Key Operational Logic” column in Table 4: This column now breaks down each mechanism (Contextual Coupling, Subject Collaboration, Evolutionary Optimization) into its detailed operational steps (e.g., perception capture → real-time decision adjustment → synchronous execution → feedback optimization).

Detailed mathematical formalization of each mechanism: As also described in our response to Reviewer #1, we have introduced formal models (MDP, optimization functions, coupling thresholds) that explicitly show how embodied intelligence transforms collaborative logic. For instance, in the Subject Collaboration Mechanism, we show how data sharing and behavioral negotiation model via a multi-agent MDP replaces the rigid, pre-programmed sequential collaboration with a dynamic, interactive, and emergent process.

**The full content is as follows:**

Table 4. Mechanisms of Embodied Intelligence-Driven Adaptive Collaboration in Supply Chains.

| **Operation Mechanism** | **Core Logic** | **Trigger Conditions** | **Key Operational Logic** | **Key Operational Links** |
| --- | --- | --- | --- | --- |
| Contextual Coupling Mechanism | Real-time matching between physical situations and digital decisions to shorten response cycles | Sudden disruptions (congestion/equipment failure), periodic fluctuations (logistics peaks), cumulative changes (increased shelf load) | 1. Perception layer captures real-time physical state $S_{perceived}(t)$; 2. Compute divergence from modeled state $S_{modeled}(t)$; 3. If divergence > threshold $\tau$, trigger reasoning layer to generate new decision $D_{new}(t)$; 4. Execution layer implements $D_{new}(t)$; 5. Feedback layer monitors execution outcomes for further refinement | Perception layer captures dynamic data → Reasoning layer dynamically adjusts decisions → Execution layer synchronously responds → Feedback layer continuously optimizes |
| Subject Collaboration Mechanism | Data sharing and behavioral negotiation among multiple embodied intelligent agents to achieve global collaborative optimization | Cross-link connection needs (warehousing-sorting-delivery linkage), resource scheduling needs (equipment/capacity allocation) | 1. Agents share real-time state data via IoT; 2. Multi-agent MDP formulates collaborative strategies; 3. Each agent executes assigned actions based on negotiated policy; 4. Execution outcomes are fed back to update the joint policy; 5. Negotiation rules iteratively refined through repeated interactions | Real-time data sharing among agents → Collaborative reasoning module formulates strategies → Multi-agent synchronous execution → Feedback optimizes negotiation rules |
| Evolutionary Optimization Mechanism | Continuous iteration of perception models and reasoning algorithms driven by closed-loop feedback to improve system intelligence levels | Data accumulation reaching thresholds, emergence of new scenario needs, unmet execution effects | 1. Collect execution feedback data $D_{feedback}$; 2. Compute loss $\mathcal{\mathcal{L}}(\Theta)$ based on feedback deviation from target; 3. Update parameters $\Theta_{new}=\Theta_{old}-\eta\nabla_{\Theta}\mathcal{\mathcal{L}}$; 4. Verify optimization effects through scenario testing; 5. If verified, solidify into standard strategy; otherwise, continue iteration | Collect execution feedback data → Iteratively optimize model parameters → Verify optimization effects → Solidify effective strategies → Iterate to adapt to new scenarios |

To formally characterize the embodied perception process, we define the multimodal perception function. Let $d_{visual}(t)$, $d_{tactile}(t)$, and $d_{spatial}(t)$ represent the raw data streams from visual, tactile, and spatial sensors at time $t$, respectively. The fused physical situational state $S_{perceived}(t)$ is generated through a fusion function ${\mathcal{\mathcal{F}}}_{fusion}$:

$$S_{perceived}(t)={\mathcal{\mathcal{F}}}_{fusion}\left( d_{visual}(t),d_{tactile}(t),d_{spatial}(t) \right)$$

The fusion function ${\mathcal{\mathcal{F}}}_{fusion}$ can be implemented through techniques such as Kalman filtering for spatial-temporal alignment or D-S evidence theory for uncertainty reasoning. This formalization clarifies how heterogeneous, real-time sensory inputs are transformed into a unified state representation that serves as the foundation for subsequent contextual reasoning processes.

To provide a rigorous algorithmic description, the contextual reasoning process is formalized as a reinforcement learning problem defined by the tuple $\langle\mathcal{S},\mathcal{A},\mathcal{P},\mathcal{\mathcal{R}},\gamma\rangle$:

State space $\mathcal{S}$: Includes physical situational data (e.g., shelf load $L_{shelf}$, road congestion rate $C_{road})$, equipment state data (e.g., robotic arm load rate $U_{arm}$, vehicle endurance $E_{vehicle})$, and order demand data (e.g., priority $P_{order}$, deadline $T_{deadline})$.

Action space $\mathcal{A}$: Includes equipment operation instructions (e.g., grasping force $F_{grasp}$, travel path adjustment $\Delta Path)$ and collaborative strategy instructions (e.g., order priority adjustment $\Delta Priority$, resource scheduling scheme $Schedule)$.

Reward function $\mathcal{\mathcal{R}}(s,a)$: Designed to maximize a weighted combination of three indicators:

$$\mathcal{\mathcal{R}}(s,a)=w_{1}\cdot R_{efficiency}+w_{2}\cdot R_{accuracy}+w_{3}\cdot R_{utilization}$$

where $R_{efficiency}$ reflects order fulfillment time reduction, $R_{accuracy}$ reflects operational error reduction (e.g., product damage rate), and $R_{utilization}$ reflects resource usage optimization (e.g., equipment idle rate), with $w_{1}+w_{2}+w_{3}=1$.

The optimal policy $\pi^{*}$ is obtained by maximizing the expected cumulative discounted reward:

$$\pi^{*}=\arg\max_{\pi}\mathbb{E}\left[ \sum_{t=0}^{T} \gamma^{t}\mathcal{\mathcal{R}}(s_{t},a_{t})\mid\pi\right]$$

This formalization ensures that the reasoning layer generates decisions that are not only context-adaptive but also systematically optimized toward global supply chain performance objectives.

To provide a rigorous characterization of how physical situations and digital decisions are coupled, we formalize the mechanism as follows.

Let $S_{perceived}(t)$ denote the real-time physical state perceived by the embodied perception layer at time $t$, and let $S_{modeled}(t)$ denote the state assumed by the existing digital model. The set of physical constraints (e.g., shelf load limits, delivery deadlines, equipment specifications) is denoted by $\mathcal{C}$.

Definition 1 (Coupling Function). The generation of a new decision $D_{new}(t)$ in response to a perceived physical state is defined by the coupling function:

$$D_{new}(t)=f_{coupling}\left( S_{perceived}(t),\mathcal{C} \right)$$

where $f_{coupling}$ integrates the current physical state with operational constraints to produce a context-adaptive decision.

Definition 2 (Triggering Condition). The re-coupling between the physical situation and digital decision is triggered when the divergence between the perceived state and the modeled state exceeds a predefined threshold $\tau$:

$$Triggerre-couplingif:\quad Div\left( S_{perceived}(t),S_{modeled}(t) \right)>\tau$$

where $Div(\cdot,\cdot)$ is a suitable divergence measure (e.g., weighted Euclidean distance over relevant state dimensions such as congestion rate, equipment load, and order priority).

This formalization captures the essential property that embodied intelligence does not operate on a fixed schedule but is event-driven, responding to meaningful discrepancies between the physical world and its digital representation. Compared to traditional models where decisions are updated based on fixed periodic cycles, this mechanism enables near-instantaneous adaptation with significantly shorter response times.

The multi-agent collaborative process is formalized using a multi-agent Markov Decision Process (MDP) framework to capture the dynamic interactions and negotiations among embodied intelligent agents.

Definition 3 (Multi-Agent Collaborative MDP). The collaboration among $N$ embodied intelligent agents is defined by the tuple:

$$\langle N,\mathcal{S},\{\mathcal{A}_{i}{\}}_{i=1}^{N},\mathcal{P},\{{\mathcal{\mathcal{R}}}_{i}{\}}_{i=1}^{N},\gamma\rangle$$

where:

$N$is the number of agents (e.g., warehouse robots, sorting robots, delivery vehicles);

$\mathcal{S}$ is the joint state space representing the combined physical and operational states of all agents and the environment;

$\mathcal{A}_{i}$ is the action space of agent $i$, including its available operations (e.g., path selection, grasping force, sorting speed);

$\mathcal{P}:\mathcal{S}\times\mathcal{A}_{1}\times\cdots\times\mathcal{A}_{N}\to\Delta(\mathcal{S})$ is the state transition probability function;

${\mathcal{\mathcal{R}}}_{i}:\mathcal{S}\times\mathcal{A}_{1}\times\cdots\times\mathcal{A}_{N}\to\mathbb{\mathbb{R}}$ is the reward function for agent $i$;

$\gamma\in[0,1)$ is the discount factor.

The collaborative objective is to find a joint policy $\boldsymbol{\pi}=(\pi_{1},\ldots,\pi_{N})$ that maximizes the global welfare:

$$\boldsymbol{\pi}^{*}=\arg\max_{\boldsymbol{\pi}} \mathbb{E}\left[ \sum_{t=0}^{T} \sum_{i=1}^{N} \gamma^{t}{\mathcal{\mathcal{R}}}_{i}(s_{t},\boldsymbol{a}_{t})\mid\boldsymbol{\pi} \right]$$

This formalization reveals how embodied intelligence fundamentally changes collaborative logic: instead of following pre-programmed sequential workflows, agents engage in dynamic, data-driven negotiation where each agent's actions are continuously adjusted based on the real-time states and behaviors of other agents. The emergence of globally optimized collaborative behavior arises from this ongoing process of shared perception, behavioral negotiation, and joint policy refinement.

The closed-loop evolutionary optimization is formalized as an iterative parameter optimization process driven by execution feedback.

Definition 4 (Feedback-Driven Parameter Optimization). Let $\Theta=(\Theta_{P},\Theta_{D})$ denote the combined parameter set, where $\Theta_{P}$ represents the parameters of the perception model (e.g., sensor fusion weights, feature extraction parameters) and $\Theta_{D}$ represents the parameters of the decision model (e.g., policy network weights, reward function coefficients). The loss function at iteration $k$ based on execution feedback data $D_{feedback}^{(k)}$ is defined as:

$$\mathcal{\mathcal{L}}(\Theta^{(k)};D_{feedback}^{(k)})=\alpha\cdot{\mathcal{\mathcal{L}}}_{efficiency}+\beta\cdot{\mathcal{\mathcal{L}}}_{accuracy}+\delta\cdot{\mathcal{\mathcal{L}}}_{stability}$$

where ${\mathcal{\mathcal{L}}}_{efficiency}$ measures deviation from target operational efficiency, ${\mathcal{\mathcal{L}}}_{accuracy}$ measures errors in execution precision, ${\mathcal{\mathcal{L}}}_{stability}$ measures system stability fluctuations, and $\alpha,\beta,\delta$ are weighting coefficients with $\alpha+\beta+\delta=1$.

The parameter update rule follows gradient-based optimization:

$$\Theta^{(k+1)}=\Theta^{(k)}-\eta\nabla_{\Theta}\mathcal{\mathcal{L}}(\Theta^{(k)};D_{feedback}^{(k)})$$

where $\eta$ is the learning rate.

This formalization captures the self-improving nature of embodied intelligent supply chains. As execution data accumulates with operational time, the iterative parameter updates lead to progressively improved performance. The convergence property:

$$\lim_{k\to\infty} \mathcal{\mathcal{L}}(\Theta^{(k)})\to{\mathcal{\mathcal{L}}}_{min}$$

represents the system's capacity for continuous intelligence evolution, where the gap between actual and optimal performance narrows over time. Compared to traditional static optimization models where parameters are set once and remain fixed, this mechanism enables the supply chain to autonomously adapt to emerging patterns, new product types, and shifting environmental conditions without manual reconfiguration.

**Comment 3**: Related research should be more comprehensive, for example, *Potential of large language models in blockchain-based supply chain finance* [J]. *Enterprise Information Systems*, 2025: 2541-199.

**Response**: We thank the reviewer for this recommendation. We have reviewed the suggested paper and found it highly relevant to the convergence of advanced AI in supply chains. We have:

Cited this work in Section 2.1.1 to enrich our discussion on the expanding landscape of AI applications.

Referenced it again in Section 7.2 (Future Research Directions: Technology Integration) to support our proposed exploration of combining Generative AI and blockchain with embodied intelligence.

**The full content is as follows:**

Furthermore, recent studies have shown that supply chain financing can alleviate the financial constraints of small and medium-sized enterprises, and blockchain technology can optimize supply chain financing strategies. These studies also point out that large language models, with their unique natural language processing capabilities, have broad application prospects in supply chain financing decision-making. Specifically, relevant research has innovatively introduced large language models to explore their application in blockchain-driven supply chain financing platforms, and found that the excellent information processing capabilities of large language models can significantly improve users’ financing efficiency. This line of research highlights the application potential of large language models in this field and provides decision-making reference for managers [22]. Such developments highlight the broader trend of AI integration across supply chain functions, yet they remain primarily focused on digital layer optimization without addressing the physical-digital integration challenge central to this study.

Explore the deep integration path of generative AI and embodied reasoning, leveraging the innovative capabilities of generative AI such as large language models to assist contextual reasoning models in generating diverse decision schemes. Recent studies explore the integration of generative AI (e.g., large language models) and embodied reasoning to generate diverse decision schemes. Research on large language models in blockchain-based supply chain finance [22] shows their integration with blockchain enhances financing efficiency and provides reliable decision support. Similarly, exploring how generative AI can augment embodied reasoning for complex unknown scenarios represents a promising research frontier for enhancing adaptability and innovative decision-making capabilities.

**Comment 4**: It is recommended to add definitions or mathematical descriptions to more rigorously characterize the collaborative mechanism, adaptability, and emergent behavior.

**Response**: This suggestion aligns perfectly with Reviewer #1’s key concern. As detailed extensively in our response to Reviewer #1 and Comment 2 above, we have integrated mathematical definitions and formal descriptions for the collaborative mechanisms (Section 5) and the framework layers (Section 4.2). These additions provide a rigorous characterization of:

Adaptability (through the coupling function and divergence threshold in Section 5.1),

Collaborative mechanism (through the multi-agent MDP in Section 5.2),

Emergent behavior (through the iterative optimization process driven by feedback in Section 5.3).

We believe these formalisms elevate the paper from a purely conceptual description to a more scientifically rigorous contribution.

**Comment 5**: The four-dimensional framework remains at the conceptual level; it is recommended to supplement it with an indicator system, algorithm flow, implementation path, etc.

**Response**: This is an excellent point that significantly improves the framework's practical value. We have made the following substantial additions:

1. Indicator System (Section 4.2, newly added Table 3): We have created a comprehensive table (Table 3) that explicitly maps each architectural layer (Embodied Perception, Contextual Reasoning, Physical Execution, Closed-Loop Feedback) to its Core Indicator Improvement Directions (e.g., Perception Accuracy, Decision Adaptability, Execution Accuracy, Model Optimization Efficiency). This table serves as an initial indicator system and connects the framework’s functions directly to measurable performance outcomes.

2. Algorithm Flow (Section 4.2.2): We have detailed the algorithm flow for the Contextual Reasoning Layer, specifying the design of the reinforcement learning-based decision engine, including the state space, action space, and reward function formulation. This moves the reasoning layer from a black-box concept to a transparent algorithmic process.

3. Implementation Path (Section 4.2 and Table 3): Table 3 also clarifies the Key Supporting Technologies (e.g., Kalman Filter, Reinforcement Learning, 5G, Edge Computing) and Typical Application Scenarios for each layer. This directly outlines a concrete implementation path.

We are confident that these revisions have transformed the framework from a conceptual model into one with clear operational, algorithmic, and evaluative dimensions.

**The full content is as follows:**

As detailed extensively in our response to Comment 2 above and Table 3.

Table 3. Correspondence between Functions, Technologies, and Applications of the Four-Dimensional Architecture of Embodied Intelligent Supply Chains.

| **Architecture Layer** | **Core Function** | **Key Supporting Technologies** | **Core Indicator Improvement Directions** | **Typical Application Scenarios** |
| --- | --- | --- | --- | --- |
| Embodied Perception Layer | Multimodal real-time collection of physical situations | Lidar + Visual Camera + Force Sensor + Kalman Filter + D-S Evidence Theory | Perception accuracy rate; Data real-time performance (ms); Multimodal fusion accuracy | Warehouse shelf load monitoring; Delivery road condition capture; Product attribute identification |
| Contextual Reasoning Layer | Dynamic adaptive decision-making and collaborative planning | Reinforcement Learning + Recurrent Neural Network + Constraint Optimization Algorithm + Convolutional Neural Network | Decision adaptability index; Collaboration efficiency (order fulfillment time); Response speed (decision generation time) | Dynamic replenishment priority adjustment; Congested path re-planning; Sorting strategy adaptation |
| Physical Execution Layer | Physical implementation of digital decisions and cross-link collaboration | Autonomous Mobile Robots + Robotic Arms + 5G Real-Time Communication + Digital-Physical Mapping | Execution accuracy rate; Operational stability; Cross-equipment collaboration success rate | Seamless connection between warehousing, sorting, and delivery; Precise product grasping; Dynamic obstacle avoidance |
| Closed-Loop Feedback Layer | Model iteration and intelligent optimization | Edge Computing + Feedback Data Preprocessing + Parameter Tuning Algorithm | Model optimization efficiency (iterations to convergence); Error reduction range; Intelligent evolution speed | Sorting error correction; Path planning model iteration; Grasping force optimization |

We hope that the above revisions and clarifications adequately address all the concerns raised. We are deeply grateful for the opportunity to improve our manuscript and look forward to your favorable consideration.

Sincerely,

Huiying Xu

(on behalf of all authors)
