# Supplementary material for: Embodied intelligence-driven adaptive collaboration in supply chains: A four-dimensional synergy framework and mechanism analysis
Source: PLoS One. 2026 Jun 9;21(6):e0351058. doi: 10.1371/journal.pone.0351058 (PMC13249223; doi:10.1371/journal.pone.0351058)
Supplement: Attachment — Submitted filename: Response_to_Reviewers_auresp_2.docx [file pone.0351058.s003.docx]

**Response to Reviewers**

Manuscript ID: PONE-D-26-10513R1

Title: Embodied intelligence-driven adaptive collaboration in supply chains: A four-dimensional synergy framework and mechanism analysis

Journal: PLOS ONE

Dear Editor and Reviewers,

We sincerely thank you for the positive evaluation of our revised manuscript and for the opportunity to further improve it through this minor revision. We have carefully addressed every point raised by the editor and both reviewers. Below, we provide a detailed, point-by-point response. All changes in the manuscript are highlighted in the “Revised Manuscript with Track Changes” file.

Sincerely,

Huiying Xu

(on behalf of all authors)

**Response to Journal Requirements**

1. Reference list review

**Response**: We have carefully reviewed our entire reference list to ensure it is complete and correct. No retracted papers were cited. All references have been verified for accuracy and formatting consistency with PLOS ONE style. One new, verifiable reference has been added to support the discussion of contextual reinforcement learning in Section 4.2.2 (Xian et al., 2024). All reference numbers have been checked for correct ordering.

**Response to Reviewer #3**

**Comment**: 1. “Department of” and follow-up organisation names in the two author units on the homepage of the manuscript homepage: “Department of Zhejiang Key...” and “Department of School of...” Connected together. Please check manually before submitting the draft to ensure that the English expression of the two units conforms to the PLOS ONE template specifications.

**Response**: We sincerely apologize for this formatting error. The author affiliations have been corrected by removing the erroneous “Department of” prefixes. The revised affiliations now read as independent institutional names in full compliance with PLOS ONE template specifications.

**The full content is as follows:**

^1^ Zhejiang Key Laboratory of Intelligent Education Technology and Application, Jinhua, China

^2^ School of Computer Science and Technology of Zhejiang Normal University, Jinhua, China

**Comment**: 2. In Section 4.2 Table 3, the author puts forward the direction of core indicators such as perception accuracy, decision-making adaptability index, execution accuracy, and model optimisation efficiency, and in Section 7.1, he takes “building a supply chain intelligent measurement index system” as an important future research direction. However, at present, this part lacks the echo of the existing “PREF (Perception–Reasoning–Execution–Feedback) performance measurement” research, which makes the proposal of the indicator system detached from the existing academic accumulation.

It is suggested that the author should supplement the citation and briefly discuss the following literature:

Faris, A., Hassan, N. A., Wei, J. L., & Othman, S. R. (2025). Measuring Embodied Adaptability in Supply Chains: A Data Analytics Model for Perception-Reasoning-Execution-Feedback Performance. Journal of Business and Data Analytics, 3(1), 78–98. https://doi.org/10.63646/jbda.2025.030105

This article proposes an adaptive data analysis and measurement model for the four links of P-R-E-F, which is highly compatible with the four-layer architecture of this article. It is recommended to indicate the connection between the indicator dimension of this article and the measurement framework proposed in the article in 1–2 sentences after Table 3 (or at the end of Section 4.2.4), and to quote it again in the indicator system outlook in Section 7.1 to enhance the continuity of future research. This supplement can significantly improve the literature support of the index part of this article.

**Response**: We greatly appreciate the reviewer's suggestion and fully agree that strengthening the connection between our indicator directions and existing performance measurement research is valuable. We made diligent efforts to locate the recommended reference (Faris et al., 2025, Journal of Business and Data Analytics, DOI: 10.63646/jbda.2025.030105) through multiple academic databases including Google Scholar, Web of Science, and direct DOI resolution. Unfortunately, the provided DOI does not resolve, and we were unable to locate any verifiable record of this publication.

However, we fully acknowledge the substantive concern behind this suggestion. To address it, we have made the following revisions without citing an unverifiable source:

Added a discussion paragraph after Table 3 (Section 4.2.4) that explicitly connects our indicator directions to established multidimensional performance measurement frameworks in the supply chain literature. This paragraph recognizes that existing research has developed metrics for responsiveness, flexibility, and operational efficiency, and positions our four-layer indicator structure as an extension that uniquely incorporates embodied physical execution and closed-loop feedback as distinct evaluative dimensions.

Revised Section 7.1 to ground the future indicator system construction in established performance measurement frameworks, emphasizing that future work should build upon existing multidimensional measurement approaches.

**The full content is as follows:**

The core indicator directions proposed in Table 3 align with broader trends in supply chain performance measurement research. Existing studies have established multidimensional frameworks for evaluating supply chain adaptability, including metrics for responsiveness, flexibility, and operational efficiency. The four-layer indicator structure proposed here extends these frameworks by explicitly incorporating embodied physical execution and closed-loop feedback as distinct evaluative dimensions, which have been largely overlooked in traditional supply chain performance measurement. Future empirical work should develop quantitative measures for each indicator direction and validate them through case studies and simulation experiments.

Construct a measurement index system for supply chain embodied intelligence, drawing upon established multidimensional performance measurement frameworks in the supply chain literature. Future work should clarify the quantification methods of core indicators such as perception accuracy, reasoning adaptability, execution collaboration degree, and feedback optimization efficiency, and design a scientifically rigorous indicator weight allocation model validated through empirical studies.

**Comment**: 3. Table 4 sets up “Key Operational Logic” and “Key Operational Links” columns at the same time, but the former is a detailed 5-step operation process, and the latter is an arrow abbreviated for the same process. The two columns of information are highly overlapping. It is recommended to compress “Key Operational Links” into a line of flowcharts and place them in the footnotes of the table, or directly merge them to the end of the “Key Operational Logic” column to avoid visual redundancy.

**Response**: We fully agree with this observation. We have merged the “Key Operational Links” content into the end of the “Key Operational Logic” column as concise summary flow descriptions, and removed the redundant separate column. Table 4 now presents a clear four-column structure without visual redundancy. Each mechanism entry concludes with a bolded Summary flow statement that captures the essential operational sequence.

**The full content is as follows:**

**Comment** 4. 1.1 The second half of the first paragraph of the section suddenly inserted a paragraph “intelligent education has also imposed new demands on supply chains...”, and the context about global supply The discussion of chain dynamics and uncertainty is more awkward, and the following text does not return to the educational scene. It is suggested that the author either delete the paragraph to maintain the focus of the discussion, or briefly deduct a sentence when leading to the meaning of the study, so that it does not become an isolated paragraph.

**Response**: We appreciate this observation. Rather than deleting the paragraph entirely, we have revised it to integrate more naturally into the broader discussion of supply chain dynamism. The revised text now uses the intelligent education example as one illustration of broader sectoral transformations that reshape supply-demand landscapes, and includes a bridging sentence that connects these domain-specific dynamics back to the macro-level disruptions discussed earlier. This revision ensures the paragraph flows coherently and does not appear as an isolated insertion.

**The full content is as follows:**

More broadly, sectoral transformations exemplify how societal shifts continuously reshape supply-demand landscapes. For instance, the rapid advancement of intelligent education has driven surging demand for online learning terminals, diversified smart teaching equipment, and sustained pressure on computing infrastructure supply. These domain-specific dynamics, combined with the macro-level disruptions discussed above, collectively underscore the necessity for supply chains to possess real-time adaptive capabilities rather than relying on static planning models.

Table 4. Mechanisms of Embodied Intelligence-Driven Adaptive Collaboration in Supply Chains.

| **Operation Mechanism** | **Core Logic** | **Trigger Conditions** | **Key Operational Logic** |
| --- | --- | --- | --- |
| Contextual Coupling Mechanism | Real-time matching between physical situations and digital decisions to shorten response cycles | Sudden disruptions (congestion/equipment failure), periodic fluctuations (logistics peaks), cumulative changes (increased shelf load) | 1. Perception layer captures real-time physical state $S_{perceived}(t)$; 2. Compute divergence from modeled state $S_{modeled}(t)$; 3. If divergence > threshold $\tau$, trigger reasoning layer to generate new decision $D_{new}(t)$; 4. Execution layer implements $D_{new}(t)$; 5. Feedback layer monitors execution outcomes for further refinement   Summary flow: Perception captures → Reasoning adjusts → Execution responds → Feedback optimizes |
| Subject Collaboration Mechanism | Data sharing and behavioral negotiation among multiple embodied intelligent agents to achieve global collaborative optimization | Cross-link connection needs (warehousing-sorting-delivery linkage), resource scheduling needs (equipment/capacity allocation) | 1. Agents share real-time state data via IoT; 2. Multi-agent MDP formulates collaborative strategies; 3. Each agent executes assigned actions based on negotiated policy; 4. Execution outcomes are fed back to update the joint policy; 5. Negotiation rules iteratively refined through repeated interactions   Summary flow: Agents share data→ Collaborative reasoning formulates → Multi-agent synchronous execution → Feedback optimizes negotiation rules |
| Evolutionary Optimization Mechanism | Continuous iteration of perception models and reasoning algorithms driven by closed-loop feedback to improve system intelligence levels | Data accumulation reaching thresholds, emergence of new scenario needs, unmet execution effects | 1. Collect execution feedback data $D_{feedback}$; 2. Compute loss $\mathcal{\mathcal{L}}(\Theta)$ based on feedback deviation from target; 3. Update parameters $\Theta_{new}=\Theta_{old}-\eta\nabla_{\Theta}\mathcal{\mathcal{L}}$; 4. Verify optimization effects through scenario testing; 5. If verified, solidify into standard strategy; otherwise, continue iteration   Summary flow: Collect feedback → Iteratively optimize → Verify effects → Solidify strategies → Adapt to new scenarios |

**Response to Reviewer #4**

**Comment**: 引用Ribeiro, M., Fernandes, D., Costa, H., & Almeida, P. (2026). Contextual Reasoning for Embodied Supply Chain Agents: Reinforcement Learning Policies from Physical State Perception to Collaborative Execution. Journal of AI Analytics and Applications, 4(1), 56-72. https://doi.org/10.63646/jaiaa.2026.040105

**Response**: We sincerely appreciate the reviewer's recommendation. We made extensive efforts to locate the suggested reference (Ribeiro et al., 2026, Journal of AI Analytics and Applications, DOI: 10.63646/jaiaa.2026.040105) through multiple academic databases including Google Scholar, Web of Science, Scopus, and direct DOI resolution. Unfortunately, the provided DOI does not resolve, and we were unable to find any verifiable record of this publication.

However, we fully agree with the reviewer's substantive point that the connection between reinforcement learning and contextual reasoning in supply chains should be better supported by existing literature. We have therefore:

Conducted a targeted literature search for genuine, peer-reviewed research on contextual reinforcement learning for supply chain management. Cited verifiable, relevant literature to strengthen the technical grounding of our contextual reasoning layer discussion in Section 4.2.2.

Cited a verifiable reference in Section 4.2.2: Xian et al. (2024), published in Expert Systems with Applications, which demonstrates how reinforcement learning agents can adapt across diverse operational environments for supply chain management. This provides rigorous technical grounding for the adaptive decision-making approach in our contextual reasoning layer.

**The full content is as follows:**

The reasoning model integrates embodied cognition theory and reinforcement learning algorithms. Recent advances in contextual reinforcement learning for supply chain management have demonstrated that agents trained with offline data and adapted online can effectively generalize across diverse operational environments [33], providing a technical foundation for the adaptive decision-making required in our framework. Specifically, the model extracts spatial situational features through convolutional neural networks, analyzes environmental temporal changes through recurrent neural networks,

33. Batsis A, Samothrakis S. Contextual reinforcement learning for supply chain management[J]. Expert Systems with Applications, 2024, 249: 123541.

We hope that the above revisions and clarifications adequately address all the concerns raised. We are deeply grateful for the opportunity to improve our manuscript and look forward to your favorable consideration.

Sincerely,

Huiying Xu

(on behalf of all authors)
