# Supplementary material for: Embodied intelligence-driven adaptive collaboration in supply chains: A four-dimensional synergy framework and mechanism analysis
Source: PLoS One. 2026 Jun 9;21(6):e0351058. doi: 10.1371/journal.pone.0351058 (PMC13249223; doi:10.1371/journal.pone.0351058)
Supplement: Peer review history

# Peer review: Decision Letter 0

**DOI:** 10.1371/journal.pone.0351058.r001

**Authors:** Yang (Jack) Lu

30 Apr 2026

PONE-D-26-10513 Embodied Intelligence-Driven Adaptive Collaboration in Supply Chains: A Four-Dimensional Synergy Framework and Mechanism Analysis PLOS One

Dear Dr. Xu,

Thank you for submitting your manuscript to PLOS ONE. After careful consideration, we feel that it has merit but does not fully meet PLOS ONE’s publication criteria as it currently stands. Therefore, we invite you to submit a revised version of the manuscript that addresses the points raised during the review process.

Please submit your revised manuscript by Jun 14 2026 11:59PM. If you will need more time than this to complete your revisions, please reply to this message or contact the journal office at plosone@plos.org. When you're ready to submit your revision, log on to https://www.editorialmanager.com/pone/ and select the 'Submissions Needing Revision' folder to locate your manuscript file.

Please include the following items when submitting your revised manuscript:

- A letter that responds to each point raised by the academic editor and reviewer(s). You should upload this letter as a separate file labeled 'Response to Reviewers'.
- A marked-up copy of your manuscript that highlights changes made to the original version. You should upload this as a separate file labeled 'Revised Manuscript with Track Changes'.
- An unmarked version of your revised paper without tracked changes. You should upload this as a separate file labeled 'Manuscript'.

If you would like to make changes to your financial disclosure, please include your updated statement in your cover letter. Guidelines for resubmitting your figure files are available below the reviewer comments at the end of this letter.

If applicable, we recommend that you deposit your laboratory protocols in protocols.io to enhance the reproducibility of your results. Protocols.io assigns your protocol its own identifier (DOI) so that it can be cited independently in the future. For instructions see: https://journals.plos.org/plosone/s/submission-guidelines#loc-laboratory-protocols. Additionally, PLOS ONE offers an option for publishing peer-reviewed Lab Protocol articles, which describe protocols hosted on protocols.io. Read more information on sharing protocols at https://plos.org/protocols?utm_medium=editorial-email&utm_source=authorletters&utm_campaign=protocols.

As the corresponding author, your ORCID iD is verified in the submission system and will appear in the published article. PLOS supports the use of ORCID, and we encourage all coauthors to register for an ORCID iD and use it as well. Please encourage your coauthors to verify their ORCID iD within the submission system before final acceptance, as unverified ORCID iDs will not appear in the published article. *Only* the individual author can complete the verification step; PLOS staff *cannot* verify ORCID iDs on behalf of authors.

We look forward to receiving your revised manuscript.

Kind regards,

Yang (Jack) Lu, PhD

Academic Editor

PLOS One

Journal Requirements:

When submitting your revision, we need you to address these additional requirements.

1. Please ensure that your manuscript meets PLOS ONE's style requirements, including those for file naming. The PLOS ONE style templates can be found at https://journals.plos.org/plosone/s/file?id=wjVg/PLOSOne_formatting_sample_main_body.pdf and https://journals.plos.org/plosone/s/file?id=ba62/PLOSOne_formatting_sample_title_authors_affiliations.pdf

2. Please note that PLOS One has specific guidelines on code sharing for submissions in which author-generated code underpins the findings in the manuscript. In these cases, we expect all author-generated code to be made available without restrictions upon publication of the work. Please review our guidelines at https://journals.plos.org/plosone/s/materials-and-software-sharing#loc-sharing-code and ensure that your code is shared in a way that follows best practice and facilitates reproducibility and reuse.

3. Thank you for stating the following financial disclosure:

[National Natural Science Foundation of China (62376252); Zhejiang Province Province-Land Synergy Program(2025SDXT004-3); Zhejiang Province Leading Geese Plan(2025C02025,2025C01056)].

Please state what role the funders took in the study.  If the funders had no role, please state: "The funders had no role in study design, data collection and analysis, decision to publish, or preparation of the manuscript."

If this statement is not correct you must amend it as needed.

Please include this amended Role of Funder statement in your cover letter; we will change the online submission form on your behalf.

4. Please remove any funding-related text from the manuscript. Funding information should not appear in any section or other areas of your manuscript. We will only publish funding information present in the Funding Statement section of the online submission form.

5. We note that your Data Availability Statement is currently as follows: [All relevant data are within the manuscript and its Supporting Information files.]

Please confirm at this time whether or not your submission contains all raw data required to replicate the results of your study. Authors must share the “minimal data set” for their submission. PLOS defines the minimal data set to consist of the data required to replicate all study findings reported in the article, as well as related metadata and methods (https://journals.plos.org/plosone/s/data-availability#loc-minimal-data-set-definition).

For example, authors should submit the following data:

- The values behind the means, standard deviations and other measures reported;

- The values used to build graphs;

- The points extracted from images for analysis.

Authors do not need to submit their entire data set if only a portion of the data was used in the reported study.

If your submission does not contain these data, please either upload them as Supporting Information files or deposit them to a stable, public repository and provide us with the relevant URLs, DOIs, or accession numbers. For a list of recommended repositories, please see https://journals.plos.org/plosone/s/recommended-repositories.

If there are ethical or legal restrictions on sharing a de-identified data set, please explain them in detail (e.g., data contain potentially sensitive information, data are owned by a third-party organization, etc.) and who has imposed them (e.g., an ethics committee). Please also provide contact information for a data access committee, ethics committee, or other institutional body to which data requests may be sent. If data are owned by a third party, please indicate how others may request data access.

6. Please update the spelling of the author's name Huiyin Xu/Huiying Xu in the manuscript and in the manuscript submission data (via Edit Submission).

If the reviewer comments include a recommendation to cite specific previously published works, please review and evaluate these publications to determine whether they are relevant and should be cited. There is no requirement to cite these works unless the editor has indicated otherwise.

[Note: HTML markup is below. Please do not edit.]

Reviewers' comments:

Reviewer's Responses to Questions

**Comments to the Author**

1. Is the manuscript technically sound, and do the data support the conclusions?

The manuscript must describe a technically sound piece of scientific research with data that supports the conclusions. Experiments must have been conducted rigorously, with appropriate controls, replication, and sample sizes. The conclusions must be drawn appropriately based on the data presented.

Reviewer #1: Yes

Reviewer #2: Partly

**********

2. Has the statistical analysis been performed appropriately and rigorously?

Reviewer #1: No

Reviewer #2: Yes

**********

3. Have the authors made all data underlying the findings in their manuscript fully available?

The PLOS Data policy requires authors to make all data underlying the findings described in their manuscript fully available without restriction, with rare exception (please refer to the Data Availability Statement in the manuscript PDF file). The data should be provided as part of the manuscript or its supporting information, or deposited to a public repository. For example, in addition to summary statistics, the data points behind means, medians and variance measures should be available. If there are restrictions on publicly sharing data—e.g. participant privacy or use of data from a third party—those must be specified.

Reviewer #1: Yes

Reviewer #2: Yes

**********

4. Is the manuscript presented in an intelligible fashion and written in standard English?

PLOS ONE does not copyedit accepted manuscripts, so the language in submitted articles must be clear, correct, and unambiguous. Any typographical or grammatical errors should be corrected at revision, so please note any specific errors here.

Reviewer #1: Yes

Reviewer #2: Yes

**********

5. Review Comments to the Author

Please use the space provided to explain your answers to the questions above. You may also include additional comments for the author, including concerns about dual publication, research ethics, or publication ethics. (Please upload your review as an attachment if it exceeds 20,000 characters)

Reviewer #1: The manuscript would benefit significantly from the inclusion of additional statistical analysis and well-defined mathematical equations to enhance its academic standardization. Incorporating appropriate statistical tools—such as descriptive statistics, correlation analysis, regression models, hypothesis testing or optimization techniques—would strengthen the reliability and validity of the research findings.

Reviewer #2: The manuscript introduces intelligence into the field of supply chain collaboration, and its topic has practical value. However, the manuscript still has several areas for improvement.

1. The theoretical and practical contributions are too general, lacking a clear comparison with existing literature to explain what this paper truly advances.

2. Existing mechanism descriptions are too macroscopic, failing to reveal how intelligence fundamentally changes the collaborative logic.

3. Related research should be more comprehensive, for example, *Potential of large language models in blockchain-based supply chain finance* [J]. *Enterprise Information Systems*, 2025: 2541-199.

4. It is recommended to add definitions or mathematical descriptions to more rigorously characterize the collaborative mechanism, adaptability, and emergent behavior.

5. The four-dimensional framework remains at the conceptual level; it is recommended to supplement it with an indicator system, algorithm flow, implementation path, etc.

**********

6. PLOS authors have the option to publish the peer review history of their article (what does this mean?). If published, this will include your full peer review and any attached files.

If you choose “no”, your identity will remain anonymous but your review may still be made public.

**Do you want your identity to be public for this peer review?** For information about this choice, including consent withdrawal, please see our Privacy Policy.

Reviewer #1: **Yes:** Dr Vipin Kumar

Reviewer #2: No

**********

[NOTE: If reviewer comments were submitted as an attachment file, they will be attached to this email and accessible via the submission site. Please log into your account, locate the manuscript record, and check for the action link "View Attachments". If this link does not appear, there are no attachment files.]

To ensure your figures meet our technical requirements, please review our figure guidelines: https://journals.plos.org/plosone/s/figures

You may also use PLOS’s free figure tool, NAAS, to help you prepare publication quality figures: https://journals.plos.org/plosone/s/figures#loc-tools-for-figure-preparation.

NAAS will assess whether your figures meet our technical requirements by comparing each figure against our figure specifications.

# Author comment: Author response to Decision Letter 1

**DOI:** 10.1371/journal.pone.0351058.r002

6 May 2026

All detailed responses have been provided in the "Response to Reviewers" document. Please note that some mathematical formulas in this message may not display correctly due to formatting limitations. We apologize for any inconvenience this may cause.

Response to Reviewers

Manuscript ID: PONE-D-26-10513

Title: Embodied intelligence-driven adaptive collaboration in supply chains: A four-dimensional synergy framework and mechanism analysis

Journal: PLOS ONE

Dear Dr. Yang (Jack) Lu,

We sincerely thank you and the two reviewers for your careful reading of our manuscript and for providing such constructive and detailed feedback. These comments have been invaluable in strengthening the rigor, clarity, and depth of our work. We have carefully revised the manuscript to address every point raised. Below, we provide a detailed, point-by-point response. All changes in the manuscript are highlighted in the “Revised Manuscript with Track Changes”s file.

Sincerely,

Huiying Xu

(on behalf of all authors)

Response to Journal Requirements

1. Please ensure that your manuscript meets PLOS ONE's style requirements, including those for file naming...

Response: We have carefully reformatted the entire manuscript according to the PLOS ONE style templates provided. This includes adjustments to the title, author, affiliations, section headings, citation format, reference list style, and file naming conventions. The revised manuscript now fully complies with all journal style requirements.

2. Please note that PLOS One has specific guidelines on code sharing for submissions in which author-generated code underpins the findings in the manuscript. In these cases, we expect all author-generated code to be made available without restrictions upon publication of the work...

Response: We fully understand the code sharing policy. Our manuscript is a theoretical study that proposes a conceptual framework and analyzes its mechanisms. No author-generated code was used to produce the findings. Should any code be developed in future follow-up empirical work, we will of course share it in accordance with PLOS ONE’s policy.

3. Please state what role the funders took in the study.

Response: We have updated the funding statement. The amended Role of Funder statement is: “The funders had no role in study design, data collection and analysis, decision to publish, or preparation of the manuscript.” Please amend the online submission form on our behalf.

4. Please remove any funding-related text from the manuscript. Funding information should not appear in any section or other areas of your manuscript. We will only publish funding information present in the Funding Statement section of the online submission form.

Response: We have completely removed all funding-related text and acknowledgements from the manuscript file. Funding information will now only appear in the Funding Statement section of the online submission form, as requested.

5. Please confirm at this time whether or not your submission contains all raw data required to replicate the results of your study...

Response: We confirm that our submission contains all data required to replicate the study findings. As this is a theoretical paper, the “minimal data set” consists of the conceptual arguments, comparative tables, and the case description presented within the manuscript. All such data are contained within the manuscript itself. No external datasets or raw data files were generated or analyzed. Should the editor require any further information, we are happy to provide it.

6. Please update the spelling of the author's name Huiyin Xu/Huiying Xu in the manuscript and in the manuscript submission data (via Edit Submission).

Response: We apologize for the inconsistency. We have now unified the spelling as Huiying Xu both in the manuscript and in the submission system, as requested.

Response to Reviewer #1

Comment: The manuscript would benefit significantly from the inclusion of additional statistical analysis and well-defined mathematical equations to enhance its academic standardization. Incorporating appropriate statistical tools—such as descriptive statistics, correlation analysis, regression models, hypothesis testing or optimization techniques—would strengthen the reliability and validity of the research findings.

Response: We are very grateful for this crucial suggestion. We fully agree that our theoretical framework needs a higher degree of formalization to meet academic standards of rigor. While the nature of our study (a theoretical framework proposal) does not involve empirical data for statistical analysis, we have substantially enhanced the manuscript by incorporating well-defined mathematical formulations and formal descriptions of our core mechanisms and architecture. The specific additions are as follows:

1. Formalization of the Embodied Perception Layer (Section 4.2.1): We have added a mathematical definition for multimodal embodied perception to characterize how raw sensor data is fused into a unified physical state. The basic principle is formalized as:

Sperceived(t)=ℱfusiondvisual(t),dtactile(t),dspatial(t)

This clarifies the transformation from raw, heterogeneous data streams to a coherent perceptual input.

2.Formalization of the Contextual Reasoning Layer (Section 4.2.2): We have detailed the reinforcement learning algorithm flow by defining the state space S, action space A, and reward function R(s,a). The decision generation is now described as a policy optimization problem:

π∗=argmaxπEt=0TγtR(st,at)

This provides a rigorous algorithmic backbone for the decision-making module.

3. Formalization of the Three Core Mechanisms (Section 5): We have introduced mathematical descriptions for each mechanism to replace purely macroscopic descriptions:

Contextual Coupling Mechanism (Section 5.1): We define a coupling function Dnew(t)=fcoupling(Sperceived(t),C) and a triggering condition Divergence(Sperceived(t),Smodeled(t))>τ. This formally captures how a discrepancy between the physical world and the digital model triggers adaptive re-decision.

Subject Collaboration Mechanism (Section 5.2): We model multi-agent collaboration using a multi-agent Markov Decision Process (MDP) with the tuple ⟨N,S,{Ai}i=1N,P,{ℛi}i=1N,γ⟩. This mathematically characterizes how multiple embodied agents interact, share data, and negotiate to achieve global optimization.

Evolutionary Optimization Mechanism (Section 5.3): We formulated the closed-loop feedback as an iterative parameter optimization problem, where a loss function ℒ(Θ) driven by execution feedback continuously updates perception and decision parameters Θ.

We believe that the addition of these mathematical formulations and algorithm descriptions significantly elevates the academic standardization of the manuscript, making the proposed framework and mechanisms both rigorous and transparent.

The full content is as follows:

To formally characterize the embodied perception process, we define the multimodal perception function. Let dvisual(t), dtactile(t), and dspatial(t) represent the raw data streams from visual, tactile, and spatial sensors at time t, respectively. The fused physical situational state Sperceived(t) is generated through a fusion function ℱfusion:

Sperceived(t)=ℱfusiondvisual(t),dtactile(t),dspatial(t)

The fusion function ℱfusion can be implemented through techniques such as Kalman filtering for spatial-temporal alignment or D-S evidence theory for uncertainty reasoning. This formalization clarifies how heterogeneous, real-time sensory inputs are transformed into a unified state representation that serves as the foundation for subsequent contextual reasoning processes.

To provide a rigorous algorithmic description, the contextual reasoning process is formalized as a reinforcement learning problem defined by the tuple ⟨S,A,P,ℛ,γ⟩:

State space S: Includes physical situational data (e.g., shelf load Lshelf, road congestion rate Croad), equipment state data (e.g., robotic arm load rate Uarm, vehicle endurance Evehicle), and order demand data (e.g., priority Porder, deadline Tdeadline).

Action space A: Includes equipment operation instructions (e.g., grasping force Fgrasp, travel path adjustment ΔPath) and collaborative strategy instructions (e.g., order priority adjustment ΔPriority, resource scheduling scheme Schedule).

Reward function ℛ(s,a): Designed to maximize a weighted combination of three indicators:

ℛ(s,a)=w1⋅Refficiency+w2⋅Raccuracy+w3⋅Rutilization

where Refficiency reflects order fulfillment time reduction, Raccuracy reflects operational error reduction (e.g., product damage rate), and Rutilization reflects resource usage optimization (e.g., equipment idle rate), with w1+w2+w3=1.

The optimal policy π∗ is obtained by maximizing the expected cumulative discounted reward:

π∗=argmaxπEt=0Tγtℛ(st,at)∣π

This formalization ensures that the reasoning layer generates decisions that are not only context-adaptive but also systematically optimized toward global supply chain performance objectives.

To provide a rigorous characterization of how physical situations and digital decisions are coupled, we formalize the mechanism as follows.

Let Sperceived(t) denote the real-time physical state perceived by the embodied perception layer at time t, and let Smodeled(t) denote the state assumed by the existing digital model. The set of physical constraints (e.g., shelf load limits, delivery deadlines, equipment specifications) is denoted by C.

Definition 1 (Coupling Function). The generation of a new decision Dnew(t) in response to a perceived physical state is defined by the coupling function:

Dnew(t)=fcouplingSperceived(t),C

where fcoupling integrates the current physical state with operational constraints to produce a context-adaptive decision.

Definition 2 (Triggering Condition). The re-coupling between the physical situation and digital decision is triggered when the divergence between the perceived state and the modeled state exceeds a predefined threshold τ:

Triggerre−couplingif: DivSperceived(t),Smodeled(t)>τ

where Div(⋅,⋅) is a suitable divergence measure (e.g., weighted Euclidean distance over relevant state dimensions such as congestion rate, equipment load, and order priority).

This formalization captures the essential property that embodied intelligence does not operate on a fixed schedule but is event-driven, responding to meaningful discrepancies between the physical world and its digital representation. Compared to traditional models where decisions are updated based on fixed periodic cycles, this mechanism enables near-instantaneous adaptation with significantly shorter response times.

The multi-agent collaborative process is formalized using a multi-agent Markov Decision Process (MDP) framework to capture the dynamic interactions and negotiations among embodied intelligent agents.

Definition 3 (Multi-Agent Collaborative MDP). The collaboration among N embodied intelligent agents is defined by the tuple:

⟨N,S,{Ai}i=1N,P,{ℛi}i=1N,γ⟩

where:

N is the number of agents (e.g., warehouse robots, sorting robots, delivery vehicles);

S is the joint state space representing the combined physical and operational states of all agents and the environment;

Ai is the action space of agent i, including its available operations (e.g., path selection, grasping force, sorting speed);

P:S×A1×⋯×AN→Δ(S) is the state transition probability function;

ℛi:S×A1×⋯×AN→ℝ is the reward function for agent i;

γ∈[0,1) is the discount factor.

The collaborative objective is to find a joint policy π=(π1,…,πN) that maximizes the global welfare:

π∗=argmaxπEt=0Ti=1Nγtℛi(st,at)∣π

This formalization reveals how embodied intelligence fundamentally changes collaborative logic: instead of following pre-programmed sequential workflows, agents engage in dynamic, data-driven negotiation where each agent's actions are continuously adjusted based on the real-time states and behaviors of other agents. The emergence of globally optimized collaborative behavior arises from this ongoing process of shared perception, behavioral negotiation, and joint policy refinement.

The closed-loop evolutionary optimization is formalized as an iterative parameter optimization process driven by execution feedback.

Definition 4 (Feedback-Driven Parameter Optimization). Let Θ=(ΘP,ΘD) denote the combined parameter set, where ΘP represents the parameters of the perception model (e.g., sensor fusion weights, feature extraction parameters) and ΘD represents the parameters of the decision model (e.g., policy network weights, reward function coefficients). The loss function at iteration k based on execution feedback data Dfeedback(k) is defined as:

ℒ(Θ(k);Dfeedback(k))=α⋅ℒefficiency+β⋅ℒaccuracy+δ⋅ℒstability

where ℒefficiency measures deviation from target operational efficiency, ℒaccuracy measures errors in execution precision, ℒstability measures system stability fluctuations, and α,β,δ are weighting coefficients with α+β+δ=1.

The parameter update rule follows gradient-based optimization:

Θ(k+1)=Θ(k)−η∇Θℒ(Θ(k);Dfeedback(k))

where η is the learning rate.

This formalization captures the self-improving nature of embodied intelligent supply chains. As execution data accumulates with operational time, the iterative parameter updates lead to progressively improved performance. The convergence property:

limk→∞ℒ(Θ(k))→ℒmin

represents the system's capacity for continuous intelligence evolution, where the gap between actual and optimal performance narrows over time. Compared to traditional static optimization models where parameters are set once and remain fixed, this mechanism enables the supply chain to autonomously adapt to emerging patterns, new product types, and shifting environmental conditions without manual reconfiguration.

Response to Reviewer #2

Comment 1: The theoretical and practical contributions are too general, lacking a clear comparison with existing literature to explain what this paper truly advances.

Response: We fully agree with this critique. We have rewritten the contribution sections (Section 1.2) to ensure every stated contribution is directly contextualized against existing research. Specifically:

In the theoretical contributions (Section 1.2.1), we now explicitly contrast our work with prior literature. For example, we state that while existing research adopts a “disembedded” model that abstracts supply chains into data for algorithm optimization [14-16], our work is the first to treat physical interaction as the source of intelligence. We also clarify that unlike studies focusing solely on digital mirroring (digital twins) or data sharing, our framework creates a closed-loop where physical execution feedback dynamically drives model evolution.

In the practical contributions (Section 1.2.2), we have specified how our framework provides an actionable guide that is distinct from current local optimization practices, helping enterprises shift from isolated link optimization to end-to-end embodied collaboration.

The full content is as follows:

First, in contrast to existing literature that treats artificial intelligence as an abstract optimization tool applied to supply chain data models [14-16], this study is among the first to systematically introduce embodied intelligence theory into the field of supply chain management. By clarifying the core concept, connotative boundaries, and characteristic dimensions of supply chain embodied intelligence, it fills the theoretical gap in intelligent supply chain collaboration for physical-digital integrated scenarios. This fundamentally shifts the research bias from a digital-above-all approach to a balanced physical-digital fusion perspective. Second, it breaks through the one-way, data-driven paradigm of traditional intelligent supply chains. While prior research has focused on information sharing or digital twin-based mirror simulation, this study constructs a bi-directional adaptive collaboration mechanism of ‘embodied perception-contextual reasoning-physical execution-closed-loop feedback’. This mechanism integrates the agent co-evolution logic of complex adaptive system theory with the contextual interaction logic of embodied cognition theory

Attachment Submitted filename: Response to Reviewers.docx

# Peer review: Decision Letter 1

**DOI:** 10.1371/journal.pone.0351058.r003

**Authors:** Yang (Jack) Lu

18 May 2026

PONE-D-26-10513R1 Embodied intelligence-driven adaptive collaboration in supply chains: A four-dimensional synergy framework and mechanism analysis PLOS One

Dear Dr. Xu,

Thank you for submitting your manuscript to PLOS ONE. After careful consideration, we feel that it has merit but does not fully meet PLOS ONE’s publication criteria as it currently stands. Therefore, we invite you to submit a revised version of the manuscript that addresses the points raised during the review process.

Please submit your revised manuscript by Jul 02 2026 11:59PM. If you will need more time than this to complete your revisions, please reply to this message or contact the journal office at plosone@plos.org. When you're ready to submit your revision, log on to https://www.editorialmanager.com/pone/ and select the 'Submissions Needing Revision' folder to locate your manuscript file.

Please include the following items when submitting your revised manuscript:

- A letter that responds to each point raised by the academic editor and reviewer(s). You should upload this letter as a separate file labeled 'Response to Reviewers'.
- A marked-up copy of your manuscript that highlights changes made to the original version. You should upload this as a separate file labeled 'Revised Manuscript with Track Changes'.
- An unmarked version of your revised paper without tracked changes. You should upload this as a separate file labeled 'Manuscript'.

If you would like to make changes to your financial disclosure, please include your updated statement in your cover letter. Guidelines for resubmitting your figure files are available below the reviewer comments at the end of this letter.

If applicable, we recommend that you deposit your laboratory protocols in protocols.io to enhance the reproducibility of your results. Protocols.io assigns your protocol its own identifier (DOI) so that it can be cited independently in the future. For instructions see: https://journals.plos.org/plosone/s/submission-guidelines#loc-laboratory-protocols. Additionally, PLOS ONE offers an option for publishing peer-reviewed Lab Protocol articles, which describe protocols hosted on protocols.io. Read more information on sharing protocols at https://plos.org/protocols?utm_medium=editorial-email&utm_source=authorletters&utm_campaign=protocols.

As the corresponding author, your ORCID iD is verified in the submission system and will appear in the published article. PLOS supports the use of ORCID, and we encourage all coauthors to register for an ORCID iD and use it as well. Please encourage your coauthors to verify their ORCID iD within the submission system before final acceptance, as unverified ORCID iDs will not appear in the published article. *Only* the individual author can complete the verification step; PLOS staff *cannot* verify ORCID iDs on behalf of authors.

We look forward to receiving your revised manuscript.

Kind regards,

Academic Editor

PLOS One

Journal Requirements:

If the reviewer comments include a recommendation to cite specific previously published works, please review and evaluate these publications to determine whether they are relevant and should be cited. There is no requirement to cite these works unless the editor has indicated otherwise.

Please review your reference list to ensure that it is complete and correct. If you have cited papers that have been retracted, please include the rationale for doing so in the manuscript text, or remove these references and replace them with relevant current references. Any changes to the reference list should be mentioned in the rebuttal letter that accompanies your revised manuscript. If you need to cite a retracted article, indicate the article’s retracted status in the References list and also include a citation and full reference for the retraction notice.

[Note: HTML markup is below. Please do not edit.]

Reviewers' comments:

Reviewer's Responses to Questions

**Comments to the Author**

1. If the authors have adequately addressed your comments raised in a previous round of review and you feel that this manuscript is now acceptable for publication, you may indicate that here to bypass the “Comments to the Author” section, enter your conflict of interest statement in the “Confidential to Editor” section, and submit your "Accept" recommendation.

Reviewer #3: (No Response)

Reviewer #4: (No Response)

**********

2. Is the manuscript technically sound, and do the data support the conclusions?

The manuscript must describe a technically sound piece of scientific research with data that supports the conclusions. Experiments must have been conducted rigorously, with appropriate controls, replication, and sample sizes. The conclusions must be drawn appropriately based on the data presented.

Reviewer #3: Partly

Reviewer #4: No

**********

3. Has the statistical analysis been performed appropriately and rigorously?

Reviewer #3: N/A

Reviewer #4: No

**********

4. Have the authors made all data underlying the findings in their manuscript fully available?

The PLOS Data policy requires authors to make all data underlying the findings described in their manuscript fully available without restriction, with rare exception (please refer to the Data Availability Statement in the manuscript PDF file). The data should be provided as part of the manuscript or its supporting information, or deposited to a public repository. For example, in addition to summary statistics, the data points behind means, medians and variance measures should be available. If there are restrictions on publicly sharing data—e.g. participant privacy or use of data from a third party—those must be specified.

Reviewer #3: Yes

Reviewer #4: Yes

**********

5. Is the manuscript presented in an intelligible fashion and written in standard English?

PLOS ONE does not copyedit accepted manuscripts, so the language in submitted articles must be clear, correct, and unambiguous. Any typographical or grammatical errors should be corrected at revision, so please note any specific errors here.

Reviewer #3: Yes

Reviewer #4: No

**********

6. Review Comments to the Author

Please use the space provided to explain your answers to the questions above. You may also include additional comments for the author, including concerns about dual publication, research ethics, or publication ethics. (Please upload your review as an attachment if it exceeds 20,000 characters)

Reviewer #3: Thanks to the author for his careful response to the previous round of opinions. The revised draft has made significant improvements in mathematical formalisation, contribution positioning and index system, and the whole is close to the publication level. However, there are still some details that need to be dealt with in the minor repair stage, and it is recommended that the author check them one by one.

1. "Department of" and follow-up organisation names in the two author units on the homepage of the manuscript homepage: "Department of Zhejiang Key..." and "Department of School of..." Connected together. Please check manually before submitting the draft to ensure that the English expression of the two units conforms to the PLOS ONE template specifications.

2. In Section 4.2 Table 3, the author puts forward the direction of core indicators such as perception accuracy, decision-making adaptability index, execution accuracy, and model optimisation efficiency, and in Section 7.1, he takes "building a supply chain intelligent measurement index system" as an important future research direction. However, at present, this part lacks the echo of the existing "PREF (Perception–Reasoning–Execution–Feedback) performance measurement" research, which makes the proposal of the indicator system detached from the existing academic accumulation.

It is suggested that the author should supplement the citation and briefly discuss the following literature:

Faris, A., Hassan, N. A., Wei, J. L., & Othman, S. R. (2025). Measuring Embodied Adaptability in Supply Chains: A Data Analytics Model for Perception-Reasoning-Execution-Feedback Performance. Journal of Business and Data Analytics, 3(1), 78–98. https://doi.org/10.63646/jbda.2025.030105

This article proposes an adaptive data analysis and measurement model for the four links of P-R-E-F, which is highly compatible with the four-layer architecture of this article. It is recommended to indicate the connection between the indicator dimension of this article and the measurement framework proposed in the article in 1–2 sentences after Table 3 (or at the end of Section 4.2.4), and to quote it again in the indicator system outlook in Section 7.1 to enhance the continuity of future research. This supplement can significantly improve the literature support of the index part of this article.

3. Table 4 sets up "Key Operational Logic" and "Key Operational Links" columns at the same time, but the former is a detailed 5-step operation process, and the latter is an arrow abbreviated for the same process. The two columns of information are highly overlapping. It is recommended to compress "Key Operational Links" into a line of flowcharts and place them in the footnotes of the table, or directly merge them to the end of the "Key Operational Logic" column to avoid visual redundancy.

4. 1.1 The second half of the first paragraph of the section suddenly inserted a paragraph "intelligent education has also imposed new demands on supply chains...", and the context about global supply The discussion of chain dynamics and uncertainty is more awkward, and the following text does not return to the educational scene. It is suggested that the author either delete the paragraph to maintain the focus of the discussion, or briefly deduct a sentence when leading to the meaning of the study, so that it does not become an isolated paragraph.

Reviewer #4: 引用Ribeiro, M., Fernandes, D., Costa, H., & Almeida, P. (2026). Contextual Reasoning for Embodied Supply Chain Agents: Reinforcement Learning Policies from Physical State Perception to Collaborative Execution. Journal of AI Analytics and Applications, 4(1), 56-72. https://doi.org/10.63646/jaiaa.2026.040105

**********

7. PLOS authors have the option to publish the peer review history of their article (what does this mean?). If published, this will include your full peer review and any attached files.

If you choose “no”, your identity will remain anonymous but your review may still be made public.

**Do you want your identity to be public for this peer review?** For information about this choice, including consent withdrawal, please see our Privacy Policy.

Reviewer #3: No

Reviewer #4: No

**********

[NOTE: If reviewer comments were submitted as an attachment file, they will be attached to this email and accessible via the submission site. Please log into your account, locate the manuscript record, and check for the action link "View Attachments". If this link does not appear, there are no attachment files.]

To ensure your figures meet our technical requirements, please review our figure guidelines: https://journals.plos.org/plosone/s/figures

You may also use PLOS’s free figure tool, NAAS, to help you prepare publication quality figures: https://journals.plos.org/plosone/s/figures#loc-tools-for-figure-preparation.

NAAS will assess whether your figures meet our technical requirements by comparing each figure against our figure specifications.

# Author comment: Author response to Decision Letter 2

**DOI:** 10.1371/journal.pone.0351058.r004

18 May 2026

Please refer to the "Response to Reviewers" file for a complete point-by-point response. We regret that certain table formatting may not render properly in this message.

Response to Reviewers

Manuscript ID: PONE-D-26-10513R1

Title: Embodied intelligence-driven adaptive collaboration in supply chains: A four-dimensional synergy framework and mechanism analysis

Journal: PLOS ONE

Dear Editor and Reviewers,

We sincerely thank you for the positive evaluation of our revised manuscript and for the opportunity to further improve it through this minor revision. We have carefully addressed every point raised by the editor and both reviewers. Below, we provide a detailed, point-by-point response. All changes in the manuscript are highlighted in the “Revised Manuscript with Track Changes” file.

Sincerely,

Huiying Xu

(on behalf of all authors)

Response to Journal Requirements

1. Reference list review

Response: We have carefully reviewed our entire reference list to ensure it is complete and correct. No retracted papers were cited. All references have been verified for accuracy and formatting consistency with PLOS ONE style. One new, verifiable reference has been added to support the discussion of contextual reinforcement learning in Section 4.2.2 (Xian et al., 2024). All reference numbers have been checked for correct ordering.

Response to Reviewer #3

Comment: 1. “Department of” and follow-up organisation names in the two author units on the homepage of the manuscript homepage: “Department of Zhejiang Key...” and “Department of School of...” Connected together. Please check manually before submitting the draft to ensure that the English expression of the two units conforms to the PLOS ONE template specifications.

Response: We sincerely apologize for this formatting error. The author affiliations have been corrected by removing the erroneous “Department of” prefixes. The revised affiliations now read as independent institutional names in full compliance with PLOS ONE template specifications.

The full content is as follows:

1 Zhejiang Key Laboratory of Intelligent Education Technology and Application, Jinhua, China

2 School of Computer Science and Technology of Zhejiang Normal University, Jinhua, China

Comment: 2. In Section 4.2 Table 3, the author puts forward the direction of core indicators such as perception accuracy, decision-making adaptability index, execution accuracy, and model optimisation efficiency, and in Section 7.1, he takes “building a supply chain intelligent measurement index system” as an important future research direction. However, at present, this part lacks the echo of the existing “PREF (Perception–Reasoning–Execution–Feedback) performance measurement” research, which makes the proposal of the indicator system detached from the existing academic accumulation.

It is suggested that the author should supplement the citation and briefly discuss the following literature:

Faris, A., Hassan, N. A., Wei, J. L., & Othman, S. R. (2025). Measuring Embodied Adaptability in Supply Chains: A Data Analytics Model for Perception-Reasoning-Execution-Feedback Performance. Journal of Business and Data Analytics, 3(1), 78–98. https://doi.org/10.63646/jbda.2025.030105

This article proposes an adaptive data analysis and measurement model for the four links of P-R-E-F, which is highly compatible with the four-layer architecture of this article. It is recommended to indicate the connection between the indicator dimension of this article and the measurement framework proposed in the article in 1–2 sentences after Table 3 (or at the end of Section 4.2.4), and to quote it again in the indicator system outlook in Section 7.1 to enhance the continuity of future research. This supplement can significantly improve the literature support of the index part of this article.

Response: We greatly appreciate the reviewer's suggestion and fully agree that strengthening the connection between our indicator directions and existing performance measurement research is valuable. We made diligent efforts to locate the recommended reference (Faris et al., 2025, Journal of Business and Data Analytics, DOI: 10.63646/jbda.2025.030105) through multiple academic databases including Google Scholar, Web of Science, and direct DOI resolution. Unfortunately, the provided DOI does not resolve, and we were unable to locate any verifiable record of this publication.

However, we fully acknowledge the substantive concern behind this suggestion. To address it, we have made the following revisions without citing an unverifiable source:

Added a discussion paragraph after Table 3 (Section 4.2.4) that explicitly connects our indicator directions to established multidimensional performance measurement frameworks in the supply chain literature. This paragraph recognizes that existing research has developed metrics for responsiveness, flexibility, and operational efficiency, and positions our four-layer indicator structure as an extension that uniquely incorporates embodied physical execution and closed-loop feedback as distinct evaluative dimensions.

Revised Section 7.1 to ground the future indicator system construction in established performance measurement frameworks, emphasizing that future work should build upon existing multidimensional measurement approaches.

The full content is as follows:

The core indicator directions proposed in Table 3 align with broader trends in supply chain performance measurement research. Existing studies have established multidimensional frameworks for evaluating supply chain adaptability, including metrics for responsiveness, flexibility, and operational efficiency. The four-layer indicator structure proposed here extends these frameworks by explicitly incorporating embodied physical execution and closed-loop feedback as distinct evaluative dimensions, which have been largely overlooked in traditional supply chain performance measurement. Future empirical work should develop quantitative measures for each indicator direction and validate them through case studies and simulation experiments.

Construct a measurement index system for supply chain embodied intelligence, drawing upon established multidimensional performance measurement frameworks in the supply chain literature. Future work should clarify the quantification methods of core indicators such as perception accuracy, reasoning adaptability, execution collaboration degree, and feedback optimization efficiency, and design a scientifically rigorous indicator weight allocation model validated through empirical studies.

Comment: 3. Table 4 sets up “Key Operational Logic” and “Key Operational Links” columns at the same time, but the former is a detailed 5-step operation process, and the latter is an arrow abbreviated for the same process. The two columns of information are highly overlapping. It is recommended to compress “Key Operational Links” into a line of flowcharts and place them in the footnotes of the table, or directly merge them to the end of the “Key Operational Logic” column to avoid visual redundancy.

Response: We fully agree with this observation. We have merged the “Key Operational Links” content into the end of the “Key Operational Logic” column as concise summary flow descriptions, and removed the redundant separate column. Table 4 now presents a clear four-column structure without visual redundancy. Each mechanism entry concludes with a bolded Summary flow statement that captures the essential operational sequence.

The full content is as follows:

Comment 4. 1.1 The second half of the first paragraph of the section suddenly inserted a paragraph “intelligent education has also imposed new demands on supply chains...”, and the context about global supply The discussion of chain dynamics and uncertainty is more awkward, and the following text does not return to the educational scene. It is suggested that the author either delete the paragraph to maintain the focus of the discussion, or briefly deduct a sentence when leading to the meaning of the study, so that it does not become an isolated paragraph.

Response: We appreciate this observation. Rather than deleting the paragraph entirely, we have revised it to integrate more naturally into the broader discussion of supply chain dynamism. The revised text now uses the intelligent education example as one illustration of broader sectoral transformations that reshape supply-demand landscapes, and includes a bridging sentence that connects these domain-specific dynamics back to the macro-level disruptions discussed earlier. This revision ensures the paragraph flows coherently and does not appear as an isolated insertion.

The full content is as follows:

More broadly, sectoral transformations exemplify how societal shifts continuously reshape supply-demand landscapes. For instance, the rapid advancement of intelligent education has driven surging demand for online learning terminals, diversified smart teaching equipment, and sustained pressure on computing infrastructure supply. These domain-specific dynamics, combined with the macro-level disruptions discussed above, collectively underscore the necessity for supply chains to possess real-time adaptive capabilities rather than relying on static planning models.

Table 4. Mechanisms of Embodied Intelligence-Driven Adaptive Collaboration in Supply Chains.

Operation Mechanism Core Logic Trigger Conditions Key Operational Logic

Contextual Coupling Mechanism Real-time matching between physical situations and digital decisions to shorten response cycles Sudden disruptions (congestion/equipment failure), periodic fluctuations (logistics peaks), cumulative changes (increased shelf load) 1.Perception layer captures real-time physical state Sperceived(t); 2. Compute divergence from modeled state Smodeled(t) ; 3. If divergence > threshold τ, trigger reasoning layer to generate new decision Dnew(t); 4. Execution layer implements Dnew(t); 5. Feedback layer monitors execution outcomes for further refinement

Summary flow: Perception captures → Reasoning adjusts → Execution responds → Feedback optimizes

Subject Collaboration Mechanism Data sharing and behavioral negotiation among multiple embodied intelligent agents to achieve global collaborative optimization Cross-link connection needs (warehousing-sorting-delivery linkage), resource scheduling needs (equipment/capacity allocation) 1.Agents share real-time state data via IoT; 2. Multi-agent MDP formulates collaborative strategies; 3. Each agent executes assigned actions based on negotiated policy; 4. Execution outcomes are fed back to update the joint policy; 5. Negotiation rules iteratively refined through repeated interactions

Summary flow: Agents share data→ Collaborative reasoning formulates → Multi-agent synchronous execution → Feedback optimizes negotiation rules

Evolutionary Optimization Mechanism Continuous iteration of perception models and reasoning algorithms driven by closed-loop feedback to improve system intelligence levels Data accumulation reaching thresholds, emergence of new scenario needs, unmet execution effects 1.Collect execution feedback data Dfeedback; 2. Compute loss ℒ(Θ) based on feedback deviation from target; 3. Update parameters Θnew=Θold−η∇Θℒ; 4. Verify optimization effects through scenario testing; 5. If verified, solidify into standard strategy; otherwise, continue iteration

Summary flow: Collect feedback → Iteratively optimize → Verify effects → Solidify strategies → Adapt to new scenarios

Response to Reviewer #4

Comment: 引用Ribeiro, M., Fernandes, D., Costa, H., & Almeida, P. (2026). Contextual Reasoning for Embodied Supply Chain Agents: Reinforcement Learning Policies from Physical State Perception to Collaborative Execution. Journal of AI Analytics and Applications, 4(1), 56-72. https://doi.org/10.63646/jaiaa.2026.040105

Response: We sincerely appreciate the reviewer's recommendation. We made extensive efforts to locate the suggested reference (Ribeiro et al., 2026, Journal of AI Analytics and Applications, DOI: 10.63646/jaiaa.2026.040105) through multiple academic databases including Google Scholar, Web of Science, Scopus, and direct DOI resolution. Unfortunately, the provided DOI does not resolve, and we were unable to find any verifiable record of this publication.

However, we fully agree with the reviewer's substantive point that the connection between reinforcement learning and contextual reasoning in supply chains should be better supported by existing literature. We have therefore:

Conducted a targeted literature search for genuine, peer-reviewed research on contextual reinforcement learning for supply chain management. Cited verifiable, relevant literature to strengthen the technical grounding of our contextual reasoning layer discussion in Section 4.2.2.

Cited a verifiable reference in Section 4.2.2: Xian et al. (2024), published in Expert Systems with Applications, which demonstrates how reinforcement learning agents can adapt across diverse operational environments for supply chain management. This provides rigorous technical grounding for the adaptive decision-making approach in our contextual reasoning layer.

The full content is as follows:

The reasoning model integrates embodied cognition theory and reinforcement learning algorithms. Recent advances in contextual reinforcement learning for supply chain management have demonstrated that agents trained with offline data and adapted online can effectively generalize across diverse operational environments [33], providing a technical foundation for the adaptive decision-making required in our framework. Specifically, the model extracts spatial situational features through convolutional neural networks, analyzes environmental temporal changes through recurrent neural networks,

33.Batsis A, Samothrakis S. Contextual reinforcement learning for supply chain management[J]. Expert Systems with Applications, 2024, 249: 123541.

We hope that the above revisions and clarifications adequately address all the concerns raised. We are deeply grateful for the opportunity to improve our manuscript and look forward to your favorable consideration.

Sincerely,

Huiying Xu

(on behalf of all authors)

Attachment Submitted filename: Response_to_Reviewers_auresp_2.docx

# Peer review: Decision Letter 2

**DOI:** 10.1371/journal.pone.0351058.r005

**Authors:** Yang (Jack) Lu

21 May 2026

Embodied intelligence-driven adaptive collaboration in supply chains: A four-dimensional synergy framework and mechanism analysis

PONE-D-26-10513R2

Dear Dr. Xu,

We’re pleased to inform you that your manuscript has been judged scientifically suitable for publication and will be formally accepted for publication once it meets all outstanding technical requirements.

Within one week, you’ll receive an e-mail detailing the required amendments. When these have been addressed, you’ll receive a formal acceptance letter and your manuscript will be scheduled for publication.

An invoice will be generated when your article is formally accepted. Please note, if your institution has a publishing partnership with PLOS and your article meets the relevant criteria, all or part of your publication costs will be covered. Please make sure your user information is up-to-date by logging into Editorial Manager at Editorial Manager® and clicking the ‘Update My Information' link at the top of the page. For questions related to billing, please contact billing support.

If your institution or institutions have a press office, please notify them about your upcoming paper to help maximize its impact. If they’ll be preparing press materials, please inform our press team as soon as possible -- no later than 48 hours after receiving the formal acceptance. Your manuscript will remain under strict press embargo until 2 pm Eastern Time on the date of publication. For more information, please contact onepress@plos.org.

Kind regards,

Yang (Jack) Lu, PhD

Academic Editor

PLOS One

Additional Editor Comments (optional):

Reviewers' comments:

Reviewer's Responses to Questions

**Comments to the Author**

1. If the authors have adequately addressed your comments raised in a previous round of review and you feel that this manuscript is now acceptable for publication, you may indicate that here to bypass the “Comments to the Author” section, enter your conflict of interest statement in the “Confidential to Editor” section, and submit your "Accept" recommendation.

Reviewer #3: All comments have been addressed

Reviewer #4: (No Response)

**********

2. Is the manuscript technically sound, and do the data support the conclusions?

The manuscript must describe a technically sound piece of scientific research with data that supports the conclusions. Experiments must have been conducted rigorously, with appropriate controls, replication, and sample sizes. The conclusions must be drawn appropriately based on the data presented.

Reviewer #3: Yes

Reviewer #4: Yes

**********

3. Has the statistical analysis been performed appropriately and rigorously?

Reviewer #3: Yes

Reviewer #4: Yes

**********

4. Have the authors made all data underlying the findings in their manuscript fully available?

The PLOS Data policy requires authors to make all data underlying the findings described in their manuscript fully available without restriction, with rare exception (please refer to the Data Availability Statement in the manuscript PDF file). The data should be provided as part of the manuscript or its supporting information, or deposited to a public repository. For example, in addition to summary statistics, the data points behind means, medians and variance measures should be available. If there are restrictions on publicly sharing data—e.g. participant privacy or use of data from a third party—those must be specified.

Reviewer #3: Yes

Reviewer #4: Yes

**********

5. Is the manuscript presented in an intelligible fashion and written in standard English?

PLOS ONE does not copyedit accepted manuscripts, so the language in submitted articles must be clear, correct, and unambiguous. Any typographical or grammatical errors should be corrected at revision, so please note any specific errors here.

Reviewer #3: Yes

Reviewer #4: Yes

**********

6. Review Comments to the Author

Please use the space provided to explain your answers to the questions above. You may also include additional comments for the author, including concerns about dual publication, research ethics, or publication ethics. (Please upload your review as an attachment if it exceeds 20,000 characters)

Reviewer #3: I have carefully reviewed the revised manuscript as well as your detailed point-by-point response to all reviewer and editorial comments. The authors have fully and appropriately addressed every raised concern, including the standardization of institutional affiliations, supplementary literature discussion and citation, rational revision of tables and structural layout, and logical optimization of the manuscript context. All revisions are well-targeted, professionally polished, and fully comply with the formatting and academic norms of PLOS ONE.

This study constructs a four-dimensional theoretical framework of embodied perception–contextual reasoning–physical execution–closed-loop feedback for supply chain adaptive collaboration, systematically clarifies the internal operation mechanisms, and verifies its practical application value with typical industrial scenarios. The research topic is innovative, the theoretical construction is rigorous, the logical framework is complete, and the literature review, mechanism analysis and future research prospects are well organized. The theoretical contributions and practical implications of this work are clear and valuable, and it has fully met the publication standards of this journal. Therefore, I am pleased to recommend acceptance for publicationand wish the authors continued achievements in related research fields.

Reviewer #4: This manuscript presents a novel and well-structured framework for embodied intelligence-driven supply chain collaboration. The theoretical analysis is solid, the logic is clear, and all my concerns have been well addressed. I recommend acceptance.

**********

7. PLOS authors have the option to publish the peer review history of their article (what does this mean?). If published, this will include your full peer review and any attached files.

If you choose “no”, your identity will remain anonymous but your review may still be made public.

**Do you want your identity to be public for this peer review?** For information about this choice, including consent withdrawal, please see our Privacy Policy.

Reviewer #3: No

Reviewer #4: No

**********

# Editor report: Acceptance letter

**DOI:** 10.1371/journal.pone.0351058.r006

**Authors:** Yang (Jack) Lu

PONE-D-26-10513R2

PLOS One

Dear Dr. Xu,

I'm pleased to inform you that your manuscript has been deemed suitable for publication in PLOS One. Congratulations! Your manuscript is now being handed over to our production team.

At this stage, our production department will prepare your paper for publication. This includes ensuring the following:

* All references, tables, and figures are properly cited

* All relevant supporting information is included in the manuscript submission,

* There are no issues that prevent the paper from being properly typeset

You will receive further instructions from the production team, including instructions on how to review your proof when it is ready. Please keep in mind that we are working through a large volume of accepted articles, so please give us a few days to review your paper and let you know the next and final steps.

Lastly, if your institution or institutions have a press office, please let them know about your upcoming paper now to help maximize its impact. If they'll be preparing press materials, please inform our press team within the next 48 hours. Your manuscript will remain under strict press embargo until 2 pm Eastern Time on the date of publication. For more information, please contact onepress@plos.org.

You will receive an invoice from PLOS for your publication fee after your manuscript has reached the completed accept phase. If you receive an email requesting payment before acceptance or for any other service, this may be a phishing scheme. Learn how to identify phishing emails and protect your accounts at https://explore.plos.org/phishing.

If we can help with anything else, please email us at customercare@plos.org.

Thank you for submitting your work to PLOS ONE and supporting open access.

Kind regards,

PLOS ONE Editorial Office Staff

on behalf of

Dr. Yang (Jack) Lu

Academic Editor

PLOS One
